# Dynamic cerebral autoregulation is preserved during orthostasis and intrathoracic pressure regulation in healthy subjects: A pilot study

**DOI:** 10.14814/phy2.16027

**Published:** 2024-04-29

**Authors:** M. Skytioti, M. Wiedmann, A. Sorteberg, L. Romundstad, Y. Hassan Ali, A. Mohammad Ayoubi, I. Zilakos, M. Elstad

**Affiliations:** ^1^ Department of Molecular Medicine, Institute of Basic Medical Sciences University of Oslo Oslo Norway; ^2^ Department of Anesthesiology Oslo University Hospital Oslo Norway; ^3^ Department of Neurosurgery Oslo University Hospital Oslo Norway; ^4^ Light Structures AS Oslo Norway

**Keywords:** cerebral autoregulation, hemodynamics, impedance threshold device, passive tilting, resistance breathing, synchronization index

## Abstract

Resistance breathing may restore cardiac output (CO) and cerebral blood flow (CBF) during hypovolemia. We assessed CBF and cerebral autoregulation (CA) during tilt, resistance breathing, and paced breathing in 10 healthy subjects. Blood velocities in the internal carotid artery (ICA), middle cerebral arteries (MCA, four subjects), and aorta were measured by Doppler ultrasound in 30° and 60° semi‐recumbent positions. ICA blood flow and CO were calculated. Arterial blood pressure (ABP, Finometer), and end‐tidal CO_2_ (ETCO_2_) were recorded. ICA blood flow response was assessed by mixed‐models regression analysis. The synchronization index (SI) for the variable pairs ABP–ICA blood velocity, ABP–MCA velocities in 0.005–0.08 Hz frequency interval was calculated as a measure of CA. Passive tilting from 30° to 60° resulted in 12% decrease in CO (*p* = 0.001); ICA blood flow tended to fall (*p* = 0.04); Resistance breathing restored CO and ICA blood flow despite a 10% ETCO_2_ drop. ETCO_2_ and CO contributed to ICA blood flow variance (adjusted *R* ^2^: 0.9, *p* < 0.0001). The median SI was low (<0.2) indicating intact CA, confirmed by surrogate date testing. The peak SI was transiently elevated during resistance breathing in the 60° position. Resistance breathing may transiently reduce CA efficiency. Paced breathing did not restore CO or ICA blood flow.

## 1 INTRODUCTION

Cerebral blood flow (CBF) is tightly regulated with several overlapping mechanisms to supply the brain with oxygen and glucose. Critical reductions in cerebral perfusion may be accompanied by syncopal symptoms and predispose to cerebral damage such as ischemic stroke. Cerebral autoregulation (CA) is a pivotal protective mechanism that compensates for changes in arterial blood pressure (ABP) and preserves CBF over a certain range of ABP, albeit inherent with large between‐subject variability (Claassen, 2016; Liu et al., 2016). The CA curve also known as Lassen's curve (Numan et al., 2014), has been widely interpreted as the CBF response to changes in ABP and used to guide clinical decisions. The original curve, however, does not include within‐subject data; instead, it was rather based on data from 11 groups of patients with varying ABP levels. A more passive relationship between CBF and ABP has been demonstrated even within the autoregulatory range (Numan et al., 2014; Skytioti et al., 2019). In addition, CA has been described as a high‐pass filter, allowing the rapid (high‐frequency) changes in ABP to be transferred to the cerebral vasculature (Claassen et al., 2009; Skytioti et al., 2018a; Zhang et al., 1998).

Dynamic CA describes the CBF response to spontaneous or abrupt ABP oscillations and evaluates the temporal aspects of CA. Several physiological processes are known to influence dynamic CA, which is characterized as a nonstationary, nonlinear phenomenon (Giller & Mueller, 2003; Panerai, 2014; Sanders et al., 2019). Arterial partial pressure of CO_2_ (PaCO_2_) is a strong regulator of CBF (Brothers et al., 2014; Claassen et al., 2007; Peebles et al., 2007) and possibly the strongest determinant of dynamic CA (McCulloch et al., 2000; Ogoh et al., 2008; Panerai, 2014). A suitable method to study and quantify dynamic CA based on spontaneous ABP oscillations is the wavelet transformation technique (Latka et al., 2005; Peng et al., 2010; Tian et al., 2016). With this approach, nonphysiological manipulations of ABP can be avoided when evaluating CA. Wavelets can handle nonlinearities in the signals and thus address the nonlinear, nonstationary, time‐scaled nature of CA (Panerai, 2014), and can provide optimal time and frequency localization by identifying the frequency of components at any given instant of time for any frequency range with high accuracy.

Cardiac output (CO) is an independent determinant of CBF, and a linear relationship between CBF and CO has been demonstrated despite unchanged mean arterial blood pressure (MAP) (Ogoh et al., 2005, 2015a; Skytioti et al., 2016, 2019). The use of intrathoracic pressure regulation, which augments the circulatory effects of the respiratory pump and enhances venous return during hypovolemia, has been shown to improve CO and cerebral perfusion (Rickards et al., 2007; Segal et al., 2013; Yannopoulos, Metzger, et al., 2006). Improved CBF and more favorable neurological outcomes have been observed in animal models of cardiac arrest where intrathoracic pressure regulation was employed (Debaty et al., 2015; Moore et al., 2017, 2021). Whether the intrathoracic pressure regulation may affect CBF and possibly CA through mechanisms other than CO augmentation has not been elucidated.

The aims of this study were (a) to quantify CA based on slow, spontaneous ABP oscillations in healthy individuals undergoing a mild orthostatic challenge similar to the first step in the mobilization of patients after neurosurgery (Karic et al., 2017), (b) to investigate whether resistance breathing, employed to restore central hemodynamics, improves CBF or alters CA, in order to assess possible future utilization in patients, (c) to investigate whether paced, slow breathing (as a simpler method of augmenting the respiratory pump and CO) has similar hemodynamic effects as resistance breathing and its impact on CA. We hypothesized that CA would not be affected in our cohort of healthy individuals. We also hypothesized that the use of intrathoracic pressure regulation would restore both CO and CBF due to augmentation of the respiratory pump during the orthostatic challenge. Furthermore, we hypothesized that harnessing the respiratory pump by slow, paced breathing would result in similar effects, improving both CO and CBF.

## 2 METHODS

Ten young healthy volunteers ([eight males] median age: 23 years [range: 19–30 years]) were recruited. All subjects gave written, informed consent to participate in the study. All procedures were performed according to the Declaration of Helsinki (World Medical Association, 2013). The study is approved by the Regional Committee for Medical and Health Research Ethics (REK SØR‐ØST, ref: 282712).

None of the subjects was a smoker or used any medication. The subjects were instructed to abstain from caffeine beverages and strenuous physical activity for 12 h prior to the experiment. They also did not eat or drink for 2 h and abstained from alcohol for 24 h before the experiment.

### 2.1 Experimental protocol

Experiments took place in a quiet room with an ambient temperature of 22–24°C. The subjects were introduced to the laboratory environment and got comfortable with the monitoring and procedures before the experiment. The subjects were trained in paced breathing with six breaths per minute (6 bpm) with the use of a tablet application. They were also trained in the use of the impedance threshold device (ITD, [ResQGARD, Advanced Circulatory Systems, Inc.]) which was used to manipulate the intrathoracic pressure. The ITD enhances the physiological effects of the respiratory pump by applying resistance during spontaneous inspiration, thus augmenting the negative intrathoracic pressure. It includes a valve that closes when the pressure within the thorax is lower than the atmospheric pressure and a second valve that opens at a preset negative intrathoracic pressure. During inspiration, the ITD augments the vacuum within the chest, increasing venous return to the heart every time the subject inspires. The ITD has a spring‐loaded diaphragm that requires a threshold pressure during inspiration to open and allow air flow. The diaphragm is moved outward during expiration, and the exhaled air moves out through the ventilation port. The manufacturer's adjustment of the spring constant determines the level of the resistance; in this study, an ITD which lowers intrathoracic pressure to minus 7 cm H_2_O was used. The ITD lowers intrathoracic pressures resembling to inspiration against a closed glottis (Convertino, 2019; Convertino et al., 2011).

The subjects lay comfortable on a bench in a semi‐recumbent (SR) position. The experimental protocol started with a 5‐min baseline period of spontaneous breathing (SB‐30°, baseline) at 30°SR position (30SR), followed by 5 min breathing with the (ITD‐30°) and 5 min paced breathing (6 bpm‐30°) in the same position. Afterward, the position was changed to 60° semi‐recumbent position (60SR) and the same procedure was followed: 5 min spontaneous breathing (SB‐60°), 5 min breathing with ITD (ITD‐60°) and 5 min paced breathing (6 bpm‐60°). A 5‐min period of recovery in the supine position was followed. We started the experiments in the 30SR position as this is a position usually adopted in the clinical setting, for example, for neurosurgical patients (Karic et al., 2017).

### 2.2 Recordings

Mean extracranial internal carotid artery (ICA) blood velocity (5 MHz probe, insonation angle: 45°, SD‐100, Vingmed Sound) and maximum aortic velocity from the suprasternal notch (2 MHz probe, insonation angle: 20°, SD‐50, Vingmed Sound) were recorded continuously by two trained operators, using Doppler ultrasound. ICA blood velocity was measured approximately 2 cm above the bifurcation of the common carotid artery in order to avoid turbulent flow (Skytioti et al., 2016). Prior to the experiment, the diameters of the right ICA at the insonation site and the rigid aortic ring were measured. The middle cerebral artery (MCA) blood velocity was also continuously recorded bilaterally in four subjects by a trained operator using transcranial Doppler ultrasound and a fixed headband with bilateral probes (Nicolet Companion III, Nicolet Vascular, USA). Noninvasive finger ABP was recorded continuously from the middle left finger positioned at heart level and mean ABP (MAP) was calculated (Finometer, Finapres Medical System, Netherlands). The finger ABP curve was calibrated and reconstructed against brachial ABP using an upper arm cuff before the recordings. The Finometer also provided pulse rate and cardiac stroke volume (SV) and CO estimates (SV_fino_, CO_fino_) from the finger ABP curve using the Modelflow algorithm (Bogert & van Lieshout, 2005). HR was calculated from the length of R–R distance in a three lead ECG. Expiratory CO_2_ was sampled continuously from the nares and recorded by a capnograph (sidestream capnograph Cap10, Medlab GmbH, Germany). All variables were sampled at 1000 Hz and transferred online to a computer running a data collection and analysis program (Labchart 8 software, Powerlab data acquisition system, ADInstruments, Australia). End‐tidal CO_2_ (ETCO_2_) and the respiratory frequency (RF) were calculated breath by breath from the capnography signal in Labchart. The cardiac stroke volume (SV_us_) and ICA blood flow per heartbeat (mL) were calculated from blood velocities and the diameters of the aortic valvular orifice (Eriksen & Walløe, 1990) and ICA (Skytioti et al., 2016), respectively, in Labchart. In addition, CO_us_ and ICA blood flow (mL/min) were calculated beat‐by‐beat from SV_us_ and ICA blood flow volume multiplied by the instantaneous HR in Labchart.

All datasets were manually inspected to assure quality of measurements. Data were downsampled to 10 Hz for subsequent statistical analysis.

### 2.3 Statistics

Medians for all variables were calculated along 3‐min periods of continuous, technically successful recordings from each 5‐min experimental state. Medians and 95% CI were calculated by Hodges–Lehmann estimates to describe the data and the Wilcoxon matched‐pairs signed‐rank test against a two‐sided alternative was used to test the difference in ICA blood flow between experimental states (StatXact, Cytel Studio 10, Cytel Inc., Cambridge, MA, USA). To test ICA blood flow and CO_us_ changes compared to baseline, the statistical significance was set to 0.01 before analysis, after correcting for multiple comparisons by the Benjamini–Hochberg Procedure for false discovery rate (Benjamini & Hochberg, 1995), using an online calculator (False Discovery Rate|Tools [carbocation.com]). In addition, we report the *p*‐values (calculated after analysis) for the changes between experimental states in other cardiovascular variables.

The ICA blood flow static response to cardiovascular changes during position change, ITD and paced breathing was modeled using linear mixed‐effects multiple regression (SAS‐JMP 12 software for Windows, SAS Institute, Cary, NC), with ICA blood flow as the dependent variable. The use of ITD, and paced breathing (dichotomous variables: Yes/No) were categorical predictors (fixed effects). The CO_us_, MAP, and ETCO_2_ were entered as continuous predictors (covariates). The choice of the variables introduced in the ICA blood flow response model was based on the physiological significance concerning CBF regulation. Due to the study's repeated‐measures design, each subject provided seven (Claassen et al., 2009) observations. Linear mixed‐effects models can account for the correlation between repeated observations from the same subject (Fitzmaurice et al., 2011), by entering subject identity as random effect in the model.

Both fixed and random effects were selected for a standard least squares fit using the restricted maximum likelihood method (Searle et al., 2008) for the estimation of fixed effects coefficients and variance component estimates for random effects. A backward selection was performed by first entering all the explanatory variables (based on their physiological significance for cerebral perfusion) in the model and consequently removing the least significant ones. Inspection of the residual plot did not reveal deviation from the assumptions of normality and homoscedasticity. To assess the goodness of fit, the adjusted *R* ^2^ (coefficient of multiple determination) was calculated. The statistical significance level was set at *p* < 0.05.

### 2.4 Wavelets

We used the wavelet transform technique, a time‐frequency method with logarithmic frequency resolution to analyze the interrelation between oscillations in ABP and ICA blood velocity, and ABP and MCA blood velocity (bilaterally), in the very low frequency (VLF) range (0.005–0.08 Hz), and quantify the dynamic CA. CA is expected to be effective below 0.08 Hz and most effective below 0.05 Hz (Claassen et al., 2016; Czosnyka & Miller, 2014). In addition, we examined the interrelation between ETCO_2_ and ICA velocity, and ETCO_2_ and MCA velocity in the same frequency range, as PaCO_2_ is an important determinant of CA. The time‐frequency analysis tools employed in our study are implemented in MATLAB® R2015b (The MathWorks Inc., MA, US) and have been developed by the Department of Physics, Lancaster university, United Kingdom (Iatsenko et al., 2013; Sheppard et al., 2012; Stefanovska et al., 1999). The Morlet mother wavelet (Bracic & Stefanovska, 1998; Morlet, 1983) was selected for the wavelet transformation. The whole experiment (35–40 min) was analyzed for each subject.

The interaction between the aforementioned variables were studied by computing the wavelet‐based phase coherence as described by Sheppard et al. (2012) and the phase synchronization index (SI) gamma (*γ*) as described by Latka et al. (2005) and Skytioti et al. (2018a). Wavelet phase coherence, a linear operation, that quantifies the linear relationship between wavelet‐transformed data. An appendix on the wavelet method can be found in a recent article from our group (Holme et al., 2023). A low value of wavelet phase coherence indicates that there is no particular phase relationship between the two oscillating variables at a specific frequency. The SI shows the phase difference variability in a system of variables. Low values of the SI indicate absence of synchronization between two signals, thus intact CA, while high values indicate increased interdependence between signals and impaired CA (Latka et al., 2005; Peng et al., 2010).

#### 2.4.1 Surrogate data testing

Surrogate data testing serves the purpose of establishing a baseline expectation for the wavelet phase coherence and the synchronization index that would occur purely by chance. This approach helps determine whether the coherence observed in the original data is statistically significant or if it could likely arise due to random variability (Porta, Gelpi, et al., 2023). In this study, we adopt the random permutation surrogates (RP) method, also known as independent identically distributed, scrambled, or shuffled surrogates, for generating surrogate datasets. The RP surrogates are crafted by shuffling or randomly permuting the order of the original data points (Lancaster et al., 2018). Despite maintaining the same mean, variance, and histogram distribution as the original signal, RP surrogates intentionally destroy any temporal structure present in the original data. This deliberate manipulation is employed to assess whether the observed data reveals temporal structure or coherence beyond what would be anticipated by chance. RP surrogates find widespread use in applications concerning cardiovascular variability analysis (Faes et al., 2004). Their versatility is particularly valuable as they accommodate non‐Gaussian distributions. The null hypothesis is formulated as follows: the observed data is fully described by independent and identically distributed (IID) random variables. Rejecting the null hypothesis in this context implies that the data exhibit temporal structure, including correlated noise. For the purpose of this study, the wavelet phase coherence and the SI between the ABP and 100 surrogates of the ICA blood velocity signal were calculated for each subject. The SI was also calculated between ETCO_2_ and 100 surrogates of MCA blood velocity signal for one subject. The surrogate threshold curves plotted in the figures correspond to a significance level *α* of 0.05.

## 3 RESULTS

All 10 subjects completed the experimental protocol successfully. In four subjects, we additionally measured bilaterally MCA blood velocities, simultaneously with ICA blood velocity. All subjects complied successfully with the resistance breathing and with slow, paced breathing. In one subject, we had to shorten by 1 min the period with paced breathing due to signs of discomfort, which most probably resulted from hypocapnia.

**FIGURE 1:**
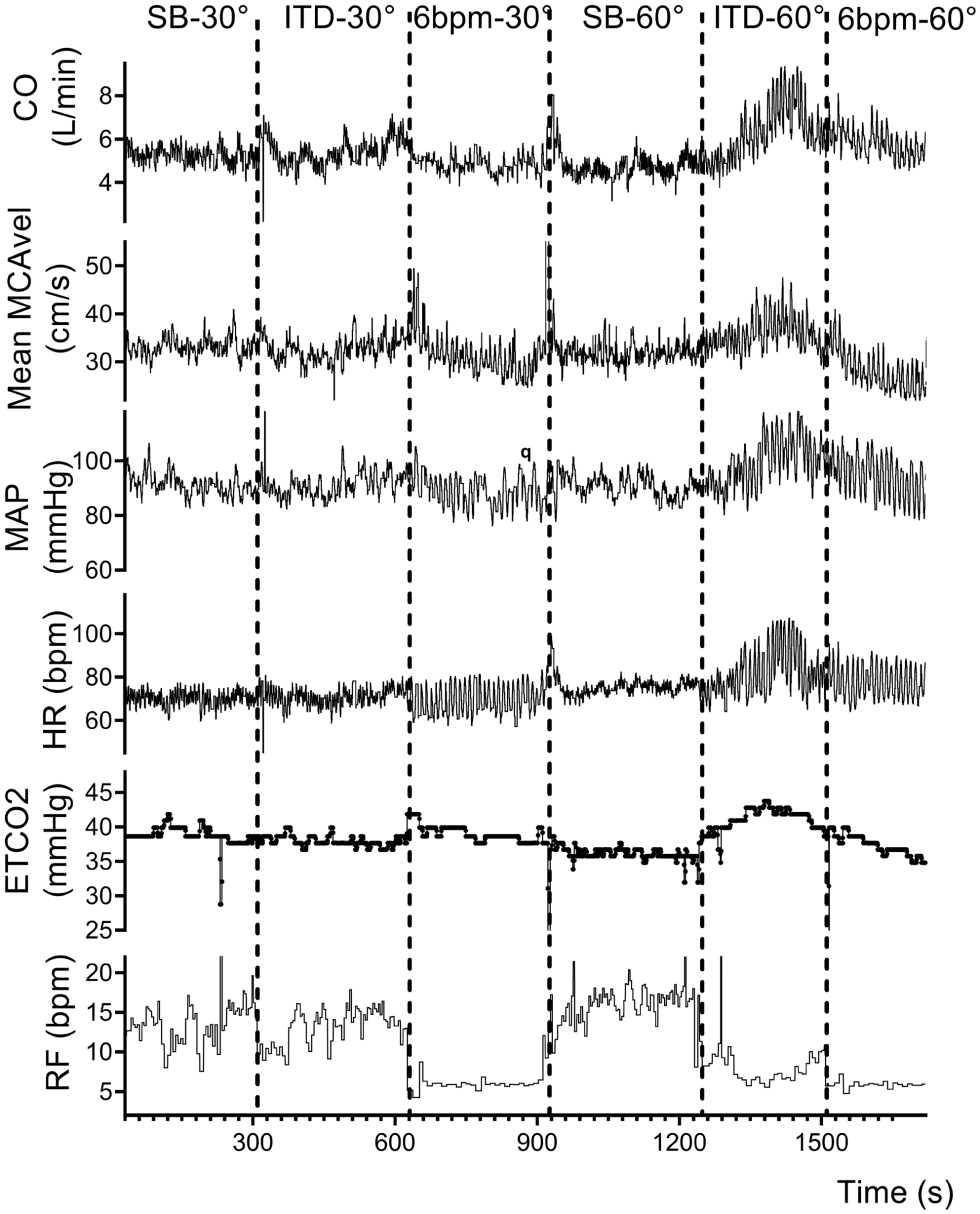
Raw recordings from one subject. 6 bpm‐30°, paced breathing with six breaths per minute in the 30° position; 6 bpm‐60°, paced breathing with six breaths per minute in the 60° position; CO, cardiac output estimate from Finometer; ETCO_2_, end‐tidal CO_2_; HR, heart rate; ITD, impedance threshold device; ITD‐30°, impedance threshold device in the 30° position; ITD‐60°, impedance threshold device in the 60° position; MAP, mean arterial blood pressure; Mean MCA Vel, averaged blood velocity in middle cerebral artery; RF, respiratory frequency; SB‐30°, spontaneous breathing in the 30° position; SB‐60°, spontaneous breathing in the 60° position.

Figure 1 shows the raw recording of cardiorespiratory and cerebrovascular variables from one subject. During ITD‐60°, CO_us_, MAP, and ETCO_2_ increased in this subject, which caused increases in MCA velocity, compared to SB‐60°. No change in either variables was observed during ITD‐30°.

### 3.1 Effects of tilting and ITD breathing

Positioning the subjects in the SB‐60° position resulted in a 12% decrease in CO_us_ (median, 95% CI: 4%–17%, *p* = 0.001) and ICA blood flow tended to decrease by 12% (median, 95% CI: 0.5%–33%, *p* = 0.04) compared to SB‐30° (Table 1). MAP and HR did not change in our cohort of healthy subjects. ETCO_2_ was reduced by about 10% after tilt and remained low at 60SR compared to 30SR (Table 1).

**TABLE 1:** Cardiorespiratory variables and internal carotid artery (ICA) blood flow changes during spontaneous breathing (SB), breathing via the ITD, and paced breathing (six breaths per minute, 6 bpm) at 30° semi‐recumbent position and at 60° semi‐recumbent position. *N* = 10.

	30° semi‐recumbent			60° semi‐recumbent		
	SB‐30°	ITD‐30°	6 bpm‐30°	SB‐60°	ITD‐60°	6 bpm‐60°
MAP (mmHg)	96 (92, 104.5)	97.5 (91, 107.5)	95.5 (89.5, 103)	98.5 (93, 105.5)	98 (91, 105)	96 (86.5, 102.5)
HR (bpm)	73 (63, 88)	71 (62, 90)	75 (59, 85)	73 (64, 87)	74 (63, 87)	74 (63, 86)
SV_fino_ (mL)	106 (75, 125)	103 (77, 128)	98 (68, 123)	92* (69, 110)	103 (82, 112)	89* (72, 112)
CO_fino_ (L/min)	7.3 (6.1, 8.7)	7.6 (6.2, 8.9)	7.0 (5.8, 8.1)	6.3* (5.4, 7.4)	7.4 (6.4, 8.6)	6.5* (5.8, 7.5)
CO_us_ (L/min)	6.9 (6, 11.2)	7.0 (6.2, 10.8)	6.8 (5.9, 10.6)	6.4* (5.6, 8.8)	7.0** (6.2, 8.9)	6.4 (5.6, 9.2)
ICAvel (m/s)	0.35 (0.33, 0.4)	0.32 (0.29, 0.36)	0.31 (0.25, 0.38)	0.32* (0.28, 0.36)	0.31* (0.28, 0.34)	0.26* (0.21, 0.31)
ICABF (mL/min)	480 (423, 933)	460 (366, 787)	427* (344, 864)	456 (360, 675)	488 (382, 656)	383* (305, 483)
ETCO_2_ (mmHg)	38.1 (36.1, 39.5)	35 (32, 39)	32.3* (30.4, 35.6)	34.4* (32.9, 36.8)	34.1* (30.7, 39.4)	30.9* (28.3,34.2)
RF (breaths/min)	14 (11, 17)	11 (8, 14)	6	14 (10, 17)	11 (7, 14)	6

*Note*: Data are medians and 95% confidence intervals calculated by Hodges–Lehmann estimates. Significance level with paired two‐sided Wilcoxon signed rank test: *p ≤ 0.01 compared to 30° spontaneous breathing.

Abbreviations: CO_fino_, cardiac output estimates from Finometer; CO_us_, cardiac output calculated from aorta velocity; ETCO_2_, End‐tidal CO_2_; HR, heart rate; ICABF, ICA blood flow; ICAvel, internal carotid artery blood velocity; ITD, impedance threshold device; MAP, mean arterial blood pressure from Finometer; RF, respiratory frequency; SB, spontaneous breathing; SV_fino_, cardiac stroke volume from Finometer.

The use of ITD in the 60SR position increased CO_us_ by 11% (median: 95% CI: 4%–20%, *p* = 0.01, Table 1) compared to spontaneous breathing. ICA blood flow was restored to baseline values (no difference between SB‐30° and ITD‐60°) during ITD‐60° despite a mild hypocapnia (Table 1).

Two different respiratory responses were observed when the subjects breathed with ITD in the 60SR position compared to SB‐60°; six subjects responded with a median increase of 4.1 mmHg in ETCO_2_, and four subjects responded with a median decrease of 5.6 mmHg in ETCO_2_. Therefore, no statistically significant change was found in ETCO_2_ during ITD compared to spontaneous breathing in the 60SR position (Table 1).

### 3.2 Effects of slow, paced breathing

**FIGURE 2:**
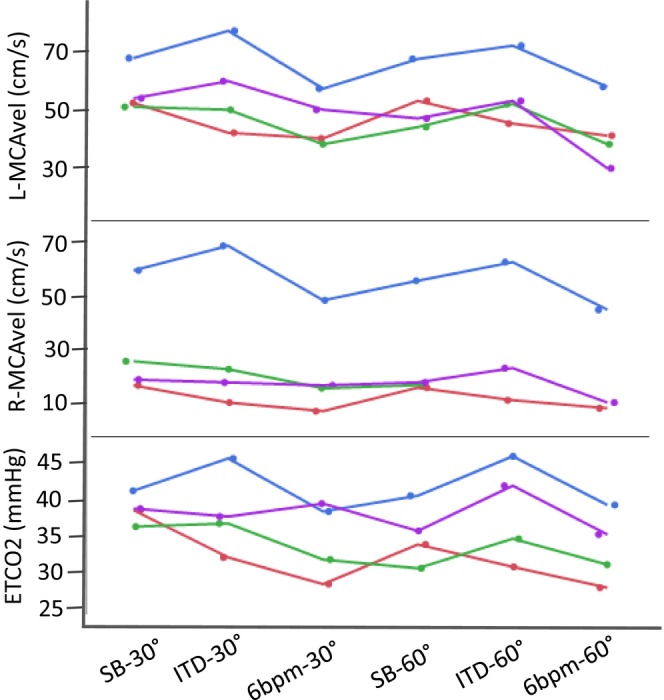
Individual responses of the blood velocities measured in the right and left middle cerebral artery (R‐MCAvel and L‐MCAvel) and of end‐tidal CO_2_ (ETCO_2_) changes at the different experimental states. 6 bpm‐30°, paced breathing with six breaths per minute in the 30° position; 6 bpm‐60°, paced breathing with six breaths per minute in the 60° position; ITD‐30°, impedance threshold device in the 30° position; ITD‐60°, impedance threshold device in the 60° position; SB‐30°, spontaneous breathing in the 30° position; SB‐60°, spontaneous breathing in the 60° position. *N* = 4.

Breathing in a paced low frequency rate (six breaths per minute) did not increase CO_us_ compared to spontaneous breathing in either 30SR or 60SR position (Table 1). ICA blood flow was decreased during slow, paced breathing both in 30SR (*p* = 0.003) and in 60SR (*p* = 0.005) position compared to spontaneous breathing (Table 1), due to the simultaneous decrease in ETCO_2_. The combined effect of this mild orthostatic challenge and slow, paced breathing with six breaths per minute was a median decrease of 29% in ICA blood flow (95% CI: 9%–46%). The blood velocities measured in MCA bilaterally in the four subjects followed closely the ETCO_2_ changes at the different experimental states and were at the lowest point during 6 bpm‐60° (Figure 2).

### 3.3 ICA blood flow prediction model

**FIGURE 3:**
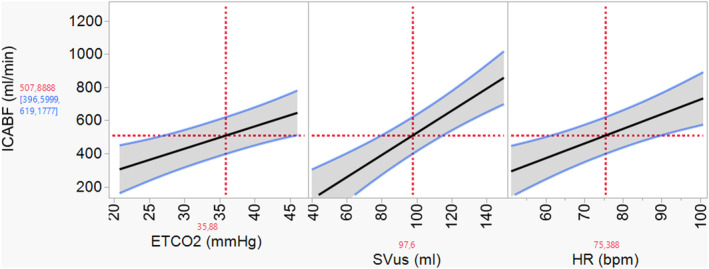
Predicted internal carotid artery blood flow (ICABF) response to changes in end‐tidal CO_2_ (ETCO_2_), cardiac stroke volume calculated from aorta velocity (SV_us_), and heart rate (HR). Solid line: mean ICABF response. Blue lines: 95% confidence intervals. Horizontal red dotted line: ICABF response at given ETCO_2_, SV_us_, and HR. Vertical red dotted lines: values of ETCO_2_, SV_us_, and HR.

The linear mixed‐effects model could explain 90% of the variance in ICA blood flow (Adjusted *R* ^2^ = 0.9, *p* < 0.0001). ETCO_2_ and CO_us_ contributed significantly to ICA blood flow variance (Model A, Table 2; Figure 3); Paced breathing, MAP, ITD (Yes/No), and Tilt (Yes/No) did not contribute to ICA blood flow variance and were therefore removed from the model. A drop of 1 L/min in CO_us_ predicted an 85 mL/min drop in ICA blood flow (*p* < 0.0001), whereas a drop of 1 mmHg in ETCO_2_ predicted a drop of 14 mL/min in ICA blood flow (*p* < 0.0001, Table 2). Of the total random variance, 65% was attributed to variability between subjects. Subsequently, CO_us_ was replaced by SV_us_ and HR in the model; both variables had a significant effect on ICA blood flow response (Model B: Adjusted *R* ^2^ = 0.88, *p* < 0.0001, Table 2; Figure 3).

**TABLE 2:** Two models A and B of the internal carotid artery blood flow response to cardiorespiratory changes.

		*β*	SE	*t* ratio	*p*‐value
A.	ETCO_2_ (mmHg)	14	3.4	4.1	<0.0001*
	CO_us_ (L/min)	85	10.7	7.9	<0.0001*
B.	ETCO_2_ (mmHg)	13.5	3.8	3.5	0.0009*
	SV_us_ (mL)	6.7	1.2	5.6	<0.0001*
	HR (bpm)	8.8	2.3	3.8	0.0005*

*Note*: Estimated regression coefficients (fixed effects) and standard errors (SE) for internal carotid artery blood flow response. In Model B: CO_us_ was replaced from SV_us_ and HR. Model A: Adjusted *R* ^2^ = 0.9, *p* < 0.0001, Model B: Adjusted *R* ^2^ = 0.88, *p* < 0.0001.

Abbreviations: CO_us_, cardiac output from ultrasound; ETCO_2_, end‐tidal CO_2_; HR (bpm), heart rate (beats per minute); SV_us_, stroke volume from ultrasound; *β*, regression coefficient.

* Statistically significant, *p* < 0.05.

### 3.4 Wavelet analysis for CA

**FIGURE 4:**
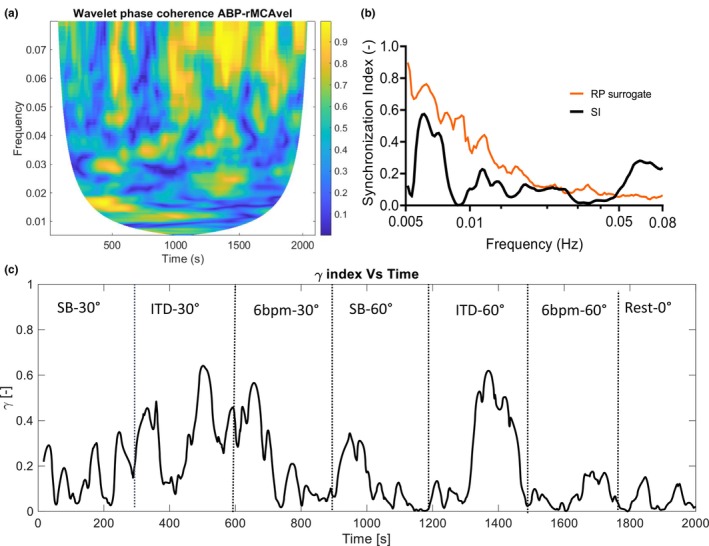
Contour plot of wavelet phase coherence (a), plot of the time‐averaged synchronization index *γ* for actual signals (black line) and for the random permutation surrogates (RP, orange line) (b), and plot of the frequency‐averaged synchronization index over the duration of the experiment (c), for the variable pair arterial blood pressure (ABP)—right middle cerebral artery blood velocity (rMCAvel). *N* = 1, same subject as in Figure 1. In this subject, ETCO_2_, CO, and MAP increased during ITD‐60°.

**FIGURE 5:**
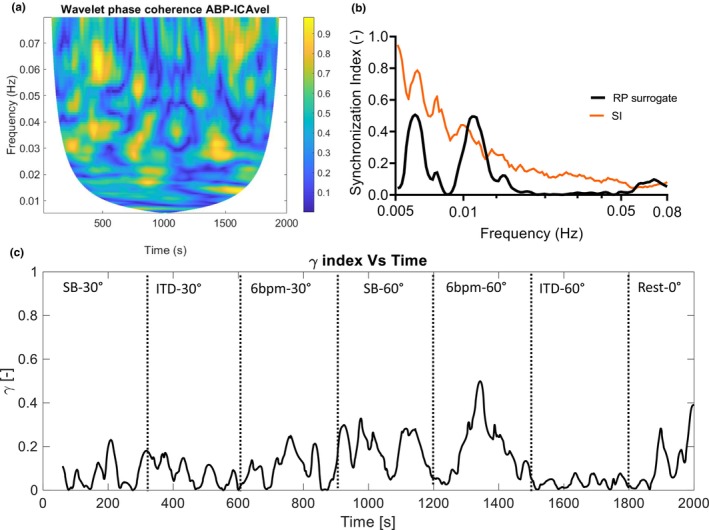
Contour plot of wavelet phase coherence (a), plot of the time‐averaged synchronization index *γ* for actual signals (black line), and for the random permutation surrogates (RP, orange line) (b), and plot of the frequency‐averaged synchronization index *γ* over the duration of the experiment (c), for the variable pair arterial blood pressure (ABP)—internal carotid artery blood velocity (ICAvel). *N* = 1 (same subject as in Figures 1 and 4). In supplementary material, similar figures from all 10 subjects can be found.

The wavelet phase coherence and the SI were calculated in the 0.005–0.08 Hz frequency interval for the variable pairs ABP—ICA velocity, ABP—right MCA velocity, and ABP—left MCA velocity (Table 3). Low values of both wavelet phase coherence and SI in the VLF interval (below 0.08 Hz) were found, indicating effective CA in all subjects. Both time‐averaged and frequency‐averaged values of the phase coherence and the SI were calculated for each subject. As shown in Table 3, similar (low) values were found for these CA indices independent of the vessel (right ICA, right MCA, or left MCA) from which velocity measurements were obtained. By frequency averaging the SI over the VLF interval (0.005–0.08 Hz) and plotting over time (Figures 4 and 5), we obtained an overview of the fluctuations in the CA effectiveness during the experiment. At ITD‐60°, the SI demonstrated a median peak of 0.35, which was significantly higher than the peak SI at baseline (Table 3). In five subjects, a peak SI over 0.35 was also observed during paced breathing, without reaching significant level.

**TABLE 3:** (A) Wavelet phase coherence and phase synchronization index (SI) for the variable pairs arterial blood pressure (ABP)—Internal carotid artery blood velocity (ICAvel), and ABP—middle cerebral artery blood velocity (MCAvel), at the very low frequency interval (0.005–0.08 Hz). (B) Peak frequency‐averaged SI over the 0.005–0.08 Hz interval in each experimental state.

A.	ABP‐ICAvel		ABP‐RMCAvel		ABP‐LMCAvel	
	Time‐averaged	Frequency‐averaged	Time‐averaged	Frequency‐averaged	Time‐averaged	Frequency‐averaged
	*N* = 10	*N* = 10	*N* = 4	*N* = 4	*N* = 4	*N* = 4
Wavelet phase coherence (−)	0.24 (0.17, 0.26)	0.42 (0.36, 0.46)	0.37 (0.21, 0.4)	0.46 (0.43, 0.48)	0.3 (0.2, 0.41)	0.49 (0.41, 0.5
SI (−)	0.06 (0.03, 0.07)	0.1 (0.07, 0.13)	0.14 (0.05, 0.16)	0.13 (0.09, 0.14)	0.1 (0.04, 0.17)	0.11 (0.09, 0.17)

B.	Peak frequency‐averaged SI					
	ABP‐ICAvel					
	SB‐30°	ITD‐30°	6 bpm‐30°	SB‐60°	ITD‐60°	6 bpm‐60°
	*N* = 10	*N* = 10	*N* = 10	*N* = 10	*N* = 10	*N* = 10
SI (−)	0.23 (0.2, 0.32)	0.32 (0.18, 0.43)	0.3 (0.22, 0.44)	0.23 (0.15, 0.4)	0.35* (0.29–0.43)	0.29 (0.24, 0.35)

*Note*: Data are medians and 95% confidence intervals, Hodges–Lehmann estimates.

Abbreviations: 6 bpm‐30°, paced breathing with six breaths/min (30° position); 6 bpm‐60°, paced breathing with six breaths/min (60° position); ITD‐30°, impedance threshold device (30° position); ITD‐60°, impedance threshold device (60° position); LMCAvel, left MCA blood velocity; *N*, number of subjects; RMCAvel, right MCA blood velocity; SB‐30°, spontaneous breathing (30° position); SB‐60°, spontaneous breathing (60° position).

* *p* = 0.01.

In Figures 4 and 5, the cold colors in the contour plots of wavelet phase coherence indicate low phase coherence and absence of synchronization between ABP and right MCA blood velocity oscillations (Figure 4a) and between ABP and ICA blood velocity oscillations (Figure 5a) in the 0.005–0.08 Hz frequency range, indicating intact CA. The time‐averaged plot of the SI over this frequency interval shows low synchronization and absence of phase locking between variables (Figures 4b and 5b). The plots of the surrogate data lie above the plot of the SI for the actual signals for the examined frequency interval, which provides a strong indication of absence of synchronization between ABP and CBF blood velocities (Figures 4b and 5b). Furthermore, the SI is averaged over the VLF frequency band, where CA is normally effective, and plotted over time for the whole experiment. The plots of the frequency‐averaged SI for the pairs ABP‐MCA blood velocity and ABP‐ICA blood velocity show low, albeit fluctuating synchronization between these variable pairs (Figures 4c and 5c). In the supplementary material, figures (similar to Figure 5) of the contour plots of the wavelet phase coherence, the time‐ and frequency‐averaged plots of the SI for the variable pair ABP‐ICA blood velocity for all subjects (Subjects 01–10) can be found.

### 3.5 Wavelet analysis to assess cerebrovascular reactivity to ETCO_2_

**FIGURE 6:**
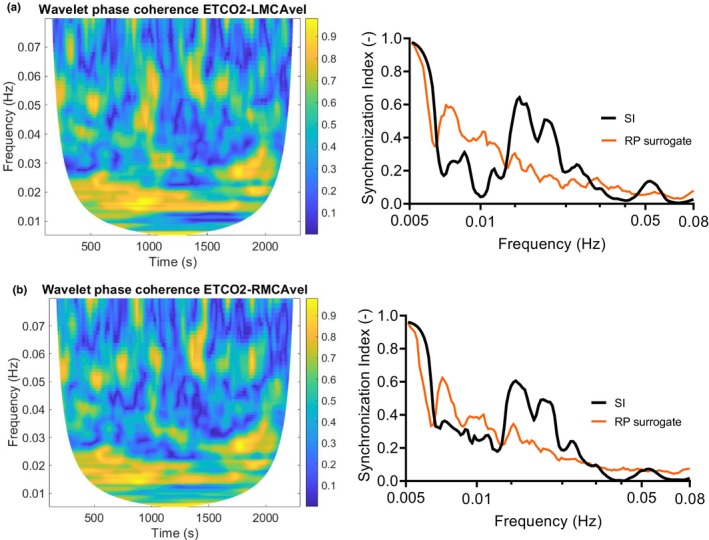
Contour plots of the wavelet phase coherence and plots of the time‐averaged gamma (*γ*) synchronization index for actual signals (black line) and for the random permutation surrogates (RP, orange line) over frequency for the variable pairs: (a) End‐tidal CO_2_ (ETCO_2_)‐left MCA velocity (LMCAvel) and (b) ETCO_2_‐right MCA velocity (RMCAvel) from one subject. Moderate values of synchronization are observed at frequencies below 0.03 Hz, indicating a degree of interrelation between MCA blood velocity and ETCO_2_.

The SI was also used to examine the interrelation between ETCO_2_ oscillations and ICA blood velocity oscillations, as well as between ETCO_2_ and MCA blood velocity oscillations in the VLF range (0.005–0.08 Hz). Moderate values of wavelet phase coherence and the SI were observed for the aforementioned variable pairs at frequencies below 0.03 Hz (Figure 6). The median peak SI for the variable pair ICA blood velocity and ETCO_2_ was 0.29 (95% CI: 0.23, 0.57). For the four subjects where the MCA blood velocities bilaterally were also measured, the median peak SI between ETCO_2_ and the right MCA velocity was 0.4 (range: 0.39–0.54). The contour plots of the wavelet phase coherence and the plots of the SI *γ* for the variables ETCO_2_‐left MCA velocity and ETCO_2_‐right MCA velocity from one subject show increased synchronization between variables at frequencies below 0.03 Hz (Figure 6). The plots of the surrogate data lie below the actual SI around 0.03 Hz in this subject confirming the increased synchronization for this variable pair (Figure 6).

## 4 DISCUSSION

We assessed the effects of intrathoracic pressure regulation and paced, slow breathing on CBF and we quantified the dynamic CA in young healthy subjects during a mild orthostatic challenge. The mild central hypovolemia, that led to a 12% CO_us_ reduction, induced by tilting the subjects from the 30SR to the 60SR position, also tended to reduce ICA blood flow (−12%) in line with previous findings (Ogoh et al., 2015b; Skytioti et al., 2016). The use of ITD in the 60SR position restored CO_us_ and ICA blood flow to baseline values despite persisting hypocapnia relative to baseline. Mixed models regression analysis showed that ETCO_2_ and CO_us_ were important determinants of ICA blood flow in agreement with previous reports (Skytioti et al., 2016, 2019). Our hypothesis that slow, paced breathing would have similar effects as ITD breathing was not confirmed. The use of the wavelet phase coherence and the SI is not widely used as a measure of CA. We found low median values of the SI during the experiments indicating effective CA. Surrogate data testing confirmed the low coherence between ABP and CBF velocities, as the plots of the SI for the surrogate data were above the plots of the SI for the actual signals. The SI showed short‐term variability indicating time‐varying dynamic CA effectiveness even in our healthy subjects. Another novel finding was that similar low values of the wavelet phase coherence and the SI were observed for the variable pairs ABP‐ICA blood velocity, ABP‐right MCA blood velocity, and ABP‐left MCA blood velocity in the four subjects in whom velocities from both ICA and MCA bilaterally were obtained. This demonstrates that the SI is a reliable measure of CA independent of the artery, MCA, or ICA, from which velocity was recorded.

### 4.1 Enhancing the respiratory pump to maintain CBF

Spontaneous respiration has beneficial effects on the circulatory homeostasis and favorable effects on the circulation have been shown in both patients and healthy individuals (Convertino et al., 2009, 2011; Lucas et al., 2013; Skytioti et al., 2018b). The negative intrathoracic pressure generated during inspiration enhances venous return and increases cardiac preload and CO (Skytioti et al., 2018b). The effects of the respiratory pump on the circulation have been indirectly demonstrated with the use of an ITD, used as a therapeutic measure in hypovolemia and hypotension (Convertino et al., 2007; Ryan et al., 2008; Yannopoulos, Metzger, et al., 2006). Breathing on the ITD has been previously shown to increase CBF velocity in resting, supine humans (Cooke et al., 2006) and in the hemorrhaging porcine model (Convertino et al., 2011). Application of negative intrathoracic pressure also reduces intracranial pressure (ICP) and subsequently elevates cerebral perfusion pressure (CPP) (Yannopoulos, McKnite, et al., 2006; Yannopoulos, Metzger, et al., 2006). We demonstrated that breathing with an ITD restored both CO and ICA blood flow after a mild orthostatic challenge in healthy individuals, in line with previous reports. All of our subjects tolerated well breathing via the ITD, although two different respiratory responses were observed: half of the subjects responded with ETCO_2_ elevation and half with ETCO_2_ reduction during ITD breathing. A relative hypocapnia, thus, was observed due to both the orthostatic challenge and resistance breathing (Table 1), which however did not prevent ICA blood flow from returning to baseline values during ITD breathing in the 60SR position. The mixed‐models regression showed that the ETCO_2_, and CO_us_ (also SV_us_ and HR) were significant predictors of ICA blood flow response during the experiments. The results were similarly independent of whether CO_fino_ or CO_us_ was used in the regression analysis. MAP was quite stable and did not contribute to ICA blood flow variance. These findings are in line with previous results from our group showing that CBF may be affected from CO changes independently of MAP (Skytioti et al., 2016).

### 4.2 Variability in dynamic CA

The efficiency of CA to buffer changes in ABP and maintain stable cerebral perfusion is influenced by several physiological mechanisms such as PaCO_2_, body temperature, the autonomic activity, neuronal activation, intracranial and intrathoracic pressure, and blood rheology (Claassen et al., 2021; Holme et al., 2023; Panerai, 2014; Panerai et al., 2010; Zhang et al., 2002). During our experiments, the median SI for the variable pairs ABP‐ICA blood velocity, ABP‐right MCA velocity, and ABP‐left MCA velocity was low (<0.2), indicating strong phase difference variability, nonstationarity (Latka et al., 2005; Panerai, 2014), and thus efficient CA in the VLF range.

The SI, however, demonstrated fluctuations with transient peaks (low phase difference variability and transient phase locking between ABP and ICA blood velocity) that momentarily reached values of 0.6 in some subjects, implying that the efficiency of CA even in healthy individuals may vary in the short term (over minutes). During the experiments, several of the aforementioned physiological determinants of CA may have contributed to fluctuations in CA efficiency. First, breathing via ITD and paced breathing induced transient changes in ETCO_2_, and consequently in PaCO_2_ (not directly measured). As PaCO_2_ is considered to be a strong determinant of CA, with hypocapnia improving and hypercapnia to reduce CA effectiveness (Dineen et al., 2010; Meel‐van den Abeelen et al., 2014; Panerai, 2014; Panerai et al., 2010), it may have been an important factor for the within‐subject CA variability observed in our experiments. Even small variations in ETCO_2_ within the normocapnic range might have induced fluctuations in CA efficiency. The low to moderate values of synchronization between ETCO_2_ and CBF velocities found at frequencies <0.03 Hz indicate some degree of interrelation between the examined variables. This finding is in line with previous reports from Mitsis and Marmarelis, that breath‐to‐breath ETCO_2_ variation is an important contributor to CBF velocity variability at frequencies <0.04 Hz (Marmarelis et al., 2016; Mitsis et al., 2004). Nonlinearity and nonstationarity characterize the relationship between ETCO_2_ and CBF velocity variations (Marmarelis et al., 2016; Mitsis et al., 2004, 2006), and therefore wavelet‐based methods are suitable to evaluate the interrelation between these variables.

Second, neuronal activation and competition between metabolic and myogenic processes during impedance breathing and paced, slow breathing may have also contributed to fluctuating CA efficiency. In a previous report that assessed the impact of transient hypocapnia and hypercapnia on dynamic CA, the latter was found to be temporarily depressed at the early stages of hyperventilation, which was maintained by metronome breathing (Dineen et al., 2010). The authors conclude that the drop in dynamic CA effectiveness might have been a response to the mental effort required during metronome pacing, and the result of increased CBF demand to meet increased brain metabolism. Impedance breathing via an ITD, as well as slow, paced breathing require both mental and physical effort (Idris et al., 2007), which both might have reduced dynamic CA efficiency and increased phase locking between ABP and CBF velocity in our study. Indeed, in eight of our subjects, transient SI peaks over 0.35 and up to 0.6 were observed during ITD breathing in the 60SR position (Figures 4 and 5). An interplay between the neuronal activation due to mental and physical effort, the variation in ETCO_2_ and the myogenic mechanisms of CA during impedance breathing might have resulted in these SI fluctuations, reducing transiently CA efficiency. Five of our subjects showed similar SI peaks also during paced breathing.

During impedance breathing, a drop in the intrathoracic pressure to at least −7 cm H_2_O was required before inspiratory airflow was allowed. How intrathoracic pressure changes may affect CBF and CA is yet unclear. Changes in ICP, PaCO_2_, and activation of autonomic reflexes may contribute. During Valsalva maneuver and an increase in the intrathoracic pressure, the CA responses are a result of both sympathetic activation elicited by the baroreflex and/or an increase in ICP (Zhang et al., 2004). The use of ganglionic blockade to abolish baroreflex‐induced sympathetic activation led to a decrease in CA efficiency and a larger drop in CBF following a marked reduction in ABP during Valsalva maneuver (Zhang et al., 2004). In a previous report, no changes in dynamic CA indices were observed in healthy normovolemic subjects during impedance breathing in the supine position despite changes in CBF velocity, ABP, and HR (Cooke et al., 2006). Likewise, in our experiments, we did not find a significant difference in peak SI during ITD breathing in the 30SR position (almost normovolemic subjects). We did however find an increase in peak SI during ITD breathing in the 60SR position, possibly due to the larger hemodynamic changes with resistance breathing during the mild central hypovolemia. Impedance breathing has been shown to decrease ICP due to increased venous drainage (Winklewski et al., 2019), which may improve CA.

The sympathetic nervous system is considered to be an important regulator of CA (Holme et al., 2023; Mitsis et al., 2009; Panerai, 2014) while a differential control of the sympathetic nervous activity in the systemic and the cerebral circulation (Ainslie et al., 2009; Cassaglia et al., 2008a, 2008b) has been suggested. An orthostatic stress that reduces CO and increases sympathetic activity has been shown to result in sympathetic cerebral vasoconstriction (Levine et al., 1994), and enhance the efficiency of CA (Holme et al., 2023). The mild orthostatic challenge in our experiments has probably resulted in sympathetic activation, following the mild drop in CO, due to the baroreflex (Charkoudian et al., 2005), and might have contributed to the observed drop in ICA blood flow and the fluctuations in CA efficiency. In individuals prone to syncope, an increased genuine information transfer from MAP to MCA blood velocity was found after a 60° passive head‐up tilt indicating impaired CA (Bari et al., 2017). These subjects were however unable to activate the cardiac arm of the baroreflex, which probably contributed to cerebral hypoperfusion during the orthostatic challenge. In our healthy subjects, the cardiac arm of the baroreflex has probably contributed to maintaining CBF at the 60SR position, as HR had a significant effect on ICA blood flow response (Table 2, Model B). In another study in which advanced nonlinear methods for evaluating coupling strength in cerebrovascular regulation were used, increased coupling strength between MAP and MCA blood velocity was also found in healthy subjects during passive tilt (Porta, Bari, et al., 2023), although this finding was probably not a sign of impaired dynamic CA (Gelpi et al., 2022). In line with these reports, we have previously shown an enhancement of ABP variability accompanied by an increase in the wavelet phase coherence between ABP and ICA blood velocity at Mayer wave frequency (around 0.1 Hz) during hypovolemia and exercise‐induced sympathetic activation in healthy subjects; however, the low values of the wavelet phase coherence and the SI over the LF interval indicated intact dynamic CA (Holme et al., 2023; Skytioti et al., 2018a). On the other hand, sympathetic nervous activity does not seem to be altered by −7 cm H_2_O of intrathoracic pressure due to impedance breathing (Winklewski et al., 2019).

### 4.3 Wavelet analysis for quantifying CA

Time‐frequency methods, such as wavelets, offer the possibility to estimate the efficiency of dynamic CA based on spontaneous oscillations in ABP and their relationship with the corresponding oscillations in CBF. The SI is a measure of the instantaneous phase difference variability between the oscillations of two signals, which was introduced recently to quantify CA. Increased values of index *γ* (and thus phase locking between the input signals) have been found in healthy subjects at the respiratory frequency (>0.15 Hz), confirming that CA cannot attenuate rapid respiration‐derived ABP oscillations (Latka et al., 2005; Skytioti et al., 2018a). In addition, the wavelet‐based SI has the advantage of locating changes in phase difference variability both in time and frequency. In this study, we could locate events of decreased phase difference variability (phase locking) between ABP and ICA/MCA blood velocity, and we observed transient SI peaks during either ITD or paced breathing.

### 4.4 Clinical implications

In critically ill patients, CBF may be compromised due to the underlying pathophysiology of the disease, hemodynamic instability, PaCO_2_ changes, and impaired CA. In particular, patients with various neurologic pathologies often experience disrupted CA and are at increased risk of cerebral hypoperfusion or hemorrhage during cardiovascular instability. Such complications can lead to poorer neurologic outcomes. Hence, comprehensive neurologic monitoring and central cardiovascular control are imperative for ensuring a favorable neurologic outcome in these patients. Monitoring and assessment of CA to individualize perfusion targets in addition to the standard cardiovascular monitoring is emerging in the critical care setting as CA seems to be an independent risk factor for worse neurological outcome (Al‐Kawaz et al., 2022; Nakano et al., 2021; Rikhraj et al., 2021). Using spontaneous low‐frequency ABP oscillations to quantify CA offers several advantages: (a) CA efficiency can be quantified and monitored bedside without the need of repeated hemodynamic challenges. This approach is not only convenient but also safer, particularly for patients. (b) The method permits continuous monitoring of CA, allowing for ongoing assessment. (c) By relying on stable physiologic conditions, this approach minimizes the influence of factors affecting CA, such as changes in autonomic activity and neuronal activation (Panerai, 2014).

The change in body position in our experiments, utilized as a mild orthostatic challenge, is often used in the clinical setting as a first step in early mobilization of neurosurgical patients. We showed that ICA blood flow was slightly reduced after positioning the subjects in the 60SR position due to a mild decrease in CO and a mild hypocapnia. The dynamic CA, calculated from spontaneous slow oscillations in ABP and both ICA and MCA blood velocity was unaffected during the challenge, although within and between subject short‐term variability in CA efficiency was observed, with transient reductions mainly during impedance breathing. In a patient population often suffering from cerebral dysregulation, the drop in CBF may be larger during passive tilting compared to our cohort of healthy volunteers.

In addition to monitoring and tightly controlling ABP in neurologic patients, monitoring of PaCO_2_ is also crucial, as it can impact both CBF and CA effectiveness. In sedated but spontaneously breathing patients, for example, increases in PaCO_2_ due to hypoventilation may impair CA and induce cerebral vasodilation and hyperemia. On the other side, mild hypocapnia is sometimes used as a temporary measure against increased ICP. Inside the normocapnic range, changes in PaCO_2_ correlate closely with changes in CBF. Wavelet analysis to quantify the interrelation between ETCO_2_ and CBF velocity showed that ETCO_2_ variations influence CBF variability at low frequencies <0.03 Hz in line with previous findings. It might be clinically relevant to determine the PaCO_2_ which is associated with the most efficient CA in the individual patient, as an optimal PaCO_2_ in addition to an optimal cerebral perfusion pressure may enhance cerebral perfusion.

### 4.5 Limitations and considerations

A main limitation of this study is that the contribution of PaCO_2_ to the fluctuations in dynamic CA efficiency was only assessed indirectly. ETCO_2_ was measured as an estimate of PaCO_2_ and the interrelation between breath‐to breath ETCO_2_ variations and CBF velocities was assessed. It has been reported that the low synchronization between ABP and CBF velocity can be partly attributed to unmeasured variability due to CO_2_ (Peng et al., 2010). We found low to moderate values of synchronization indicating some degree of interrelation between ABP and CBF at frequencies below 0.03 Hz.

In two of our subjects, the blood velocities obtained from the right MCA were rather low compared to the blood velocities obtained from the left MCA. This can be probably attributed to a suboptimal angle of insonation, as during transcranial Doppler velocity measurements, it is important to keep the angle of insonation below 30° and as close to zero as possible to minimize the Doppler shift measurement error (Purkayastha & Sorond, 2012). During the experiments, this error was however constant as the probes were firmly attached to the cranium by a fixed headband.

The order of the breathing sessions was not randomized in either position, is another limitation in this study. We decided to start always with spontaneous breathing and proceed to ITD breathing and last to paced breathing. In a repeated measures study design, the order of the experimental states may affect outcome due to learning. If the experiments started with ITD or paced breathing, the respiratory pattern during spontaneous breathing might be similar to the respiratory pattern during ITD and pacing, affecting probably the values of ETCO_2_ as well.

We quantified dynamic CA based on ABP and velocity measurements from the ICA (10 subjects), and the MCA bilaterally (four subjects). Although we found similar values of coherence and synchronization (Table 2), it is a limitation of this study that MCA velocity could not be obtained from all 10 subjects.

In a patient population with cerebral dysregulation, assessment of CA by wavelets and synchronization analysis seems promising but not adequately evaluated. The fact that increased values of the SI indicate impaired CA has been indirectly shown by calculating the SI in the low frequency (0.08–0.15 Hz) and the high frequency (0.15–0.4 Hz) intervals (where CA is not effective) in small cohorts of healthy volunteers (Holme et al., 2023; Skytioti et al., 2018a). A recent report has also evaluated the reliability of several CA indices in a group of stroke patients and concluded that the SI has good reliability and reproducibility as a CA metric (Lee et al., 2020).

A small group of only 10 young healthy subjects was recruited. However, the repeated measures design (where the subjects contribute with multiple observations and act as their own controls) increases statistical power and reduces the number of subjects needed to demonstrate an effect (Guo et al., 2013). The sample size in this study was based on previous repeated measures studies on cerebral blood flow regulation in quite homogenous cohorts of young healthy subjects from our group (Skytioti et al., 2016, 2018a). Nonparametric statistics were used as a robust statistical method for small sample sizes (Hollander & Wolfe, 1999).

Another limitation of this study is that the ICA diameter was only measured once prior to the experiment. Small, untracked changes in vessel diameter during the experiment might have affected the calculation of ICA blood flow, as ICA constriction would result in overestimation of ICA blood flow and dilation in underestimation. However, it has been previously shown that a 5% change in ICA diameter accompanied a 20% reduction in MAP (Lewis et al., 2015), and no change in ICA diameter was found during lower body negative pressure of −35 mmHg and unchanged MAP (Ogoh et al., 2015a). In this study, we observed minimal changes in MAP (Table 1) and the observed CO reduction was only 12%. Therefore, we assume that the ICA diameter remained stable during the experiments and did not contribute to the observed changes in ICA blood flow.

Last, an important consideration is that we have chosen to use wavelet analysis to quantify CA in this study and not the transfer function which still remains one of the most popular analytical techniques for the characterization CA effectiveness. The main reason for our choice is that wavelet‐based methods do not suffer from the deficiencies of the Fourier‐based methods, emanating from the underlying assumption of stationarity of the input signals; nonstationarity is an intrinsic characteristic of signals related to CA (Fan et al., 2021). Wavelet analysis also provides a frame of much finer time‐frequency resolution than the Fourier‐based methods, making no assumption about the stationarity of the analyzing input signals while offering the benefit of showing high resolution in both high and low frequency components (Shiogai et al., 2010). Wavelet transform is considered, in general, more suited to decoupling intermittent, transient, and aperiodic signal components compared to other methods (Addison, 2015).

## 5 CONCLUSION

CBF and dynamic CA were assessed by wavelet‐based SI in healthy subjects during a mild orthostatic challenge, resistance breathing, and slow, paced breathing. We showed that resistance breathing restored CO and ICA blood flow during mild hypovolemia despite a persisting mild hypocapnia. Low median SI values were observed during the experiment, indicating effective dynamic CA at frequencies below 0.08 Hz (less than five cycles/min). The SI could be used to locate episodes of reduced CA efficiency both in time and in frequency. Resistance breathing after passive tilting increased transiently the peak SI compared to baseline. ETCO_2_ was an important predictor of ICA blood flow variance and may have also contributed to the variability in dynamic CA at frequencies below 0.03 Hz.

## AUTHOR CONTRIBUTIONS

**M. Skytioti, M. Wiedmann, and M. Elstad**: Conceived and designed research; **M. Skytioti, M. Elstad, A. Mohammad Ayoubi, Y. Hassan Ali, A. Sorteberg, and L. Romundstad**: Performed experiments; **M. Skytioti and I. Zilakos**: Analyzed data; **I. Zilakos**: Performed wavelet analysis and surrogate data testing; **M. Skytioti, M. Elstad, M. Wiedmann, A. Sorteberg, L. Romundstad, and I. Zilakos**: Interpreted results of experiments; **M. Skytioti and I. Zilakos**: Prepared figures; **M. Skytioti and I. Zilakos**: Drafted article; **M. Skytioti, M. Elstad, M. Wiedmann, A. Sorteberg, L. Romundstad, and I. Zilakos**: Edited and revised article; **All**: Approved final version of article.

## FUNDING INFORMATION

No funding information provided.

## CONFLICT OF INTEREST STATEMENT

We confirm that none of the authors has any conflicts of interests.

## ETHICS STATEMENT

All subjects gave written, informed consent prior to their participation in the study, all procedures were according to the Declaration of Helsinki and the study is approved by the Regional Committee for Medical and Health Research Ethics.

## Supporting information


Data S1:

## Data Availability

The authors confirm that the data supporting the results in this article are available upon request.
