# Supplementary material for: Dynamic cerebral autoregulation is preserved during orthostasis and intrathoracic pressure regulation in healthy subjects: A pilot study
Source: Physiol Rep. 2024 Apr 29;12(9):e16027. doi: 10.14814/phy2.16027 (PMC11058003; doi:10.14814/phy2.16027)
Supplement: Supplementary file 1 — Data S1: [file PHY2-12-e16027-s001.zip › PHYSREP-2024-01-021-T-f07-z-.pdf]

Supplementary material, N=10

A. Contour plots of the wavelet phase coherence between arterial blood pressure (ABP) and internal carotid artery blood velocity (ICAvel)
B. Plots of the time-averaged synchronization index (SI) γ (-) for the variable pair ABP-ICAvel (Black line) over frequency . Plots of the Random Permutation (RP) surrogate with significance level a=0.05 (Orange line). The RP plots are above the plots of the SI in all subjects, confirming the absence of synchronization between ABP and ICAvel signals, thus effective cerebral autoregulation.
C. Plots of the frequency-averaged synchronization index γ over time for the duration of the experiment for the variable pair ABP-ICAvel.

Subject 01

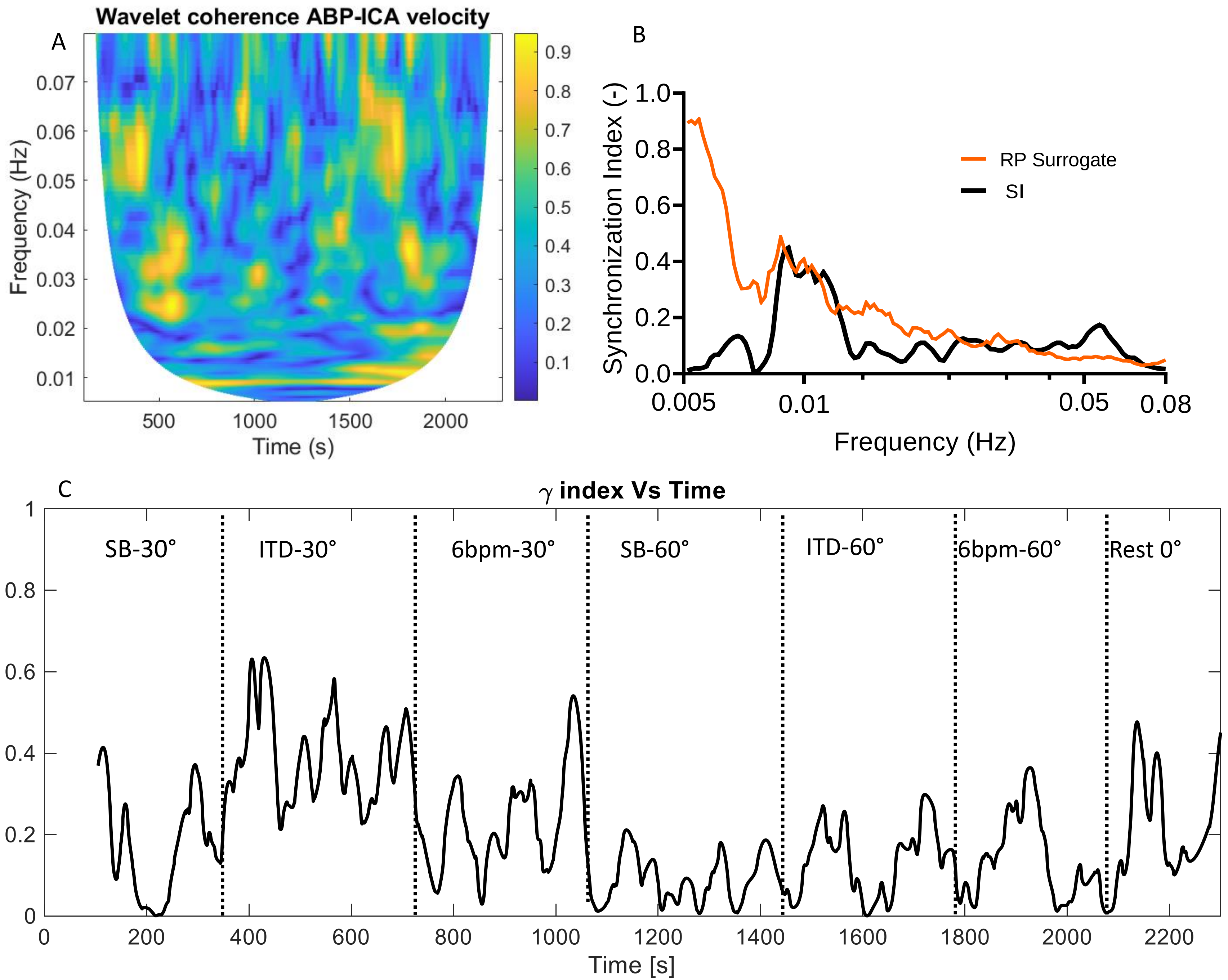

A
Wavelet coherence ABP-ICA velocity
Frequency (Hz)
0.07
0.06
0.05
0.04
0.03
0.02
0.01
500
1000
1500
2000
Time (s)
0.9
0.8
0.7
0.6
0.5
0.4
0.3
0.2
0.1
B
Synchronization Index (-)
1.0
0.8
0.6
0.4
0.2
0.0
0.005
0.01
0.05
0.08
Frequency (Hz)
RP Surrogate
SI
C
γ index Vs Time
γ [-]
SB-30°
ITD-30°
6bpm-30°
SB-60°
ITD-60°
6bpm-60°
Rest 0°
1
0.8
0.6
0.4
0.2
0
0
200
400
600
800
1000
1200
1400
1600
1800
2000
2200
Time [s]

Subject 02

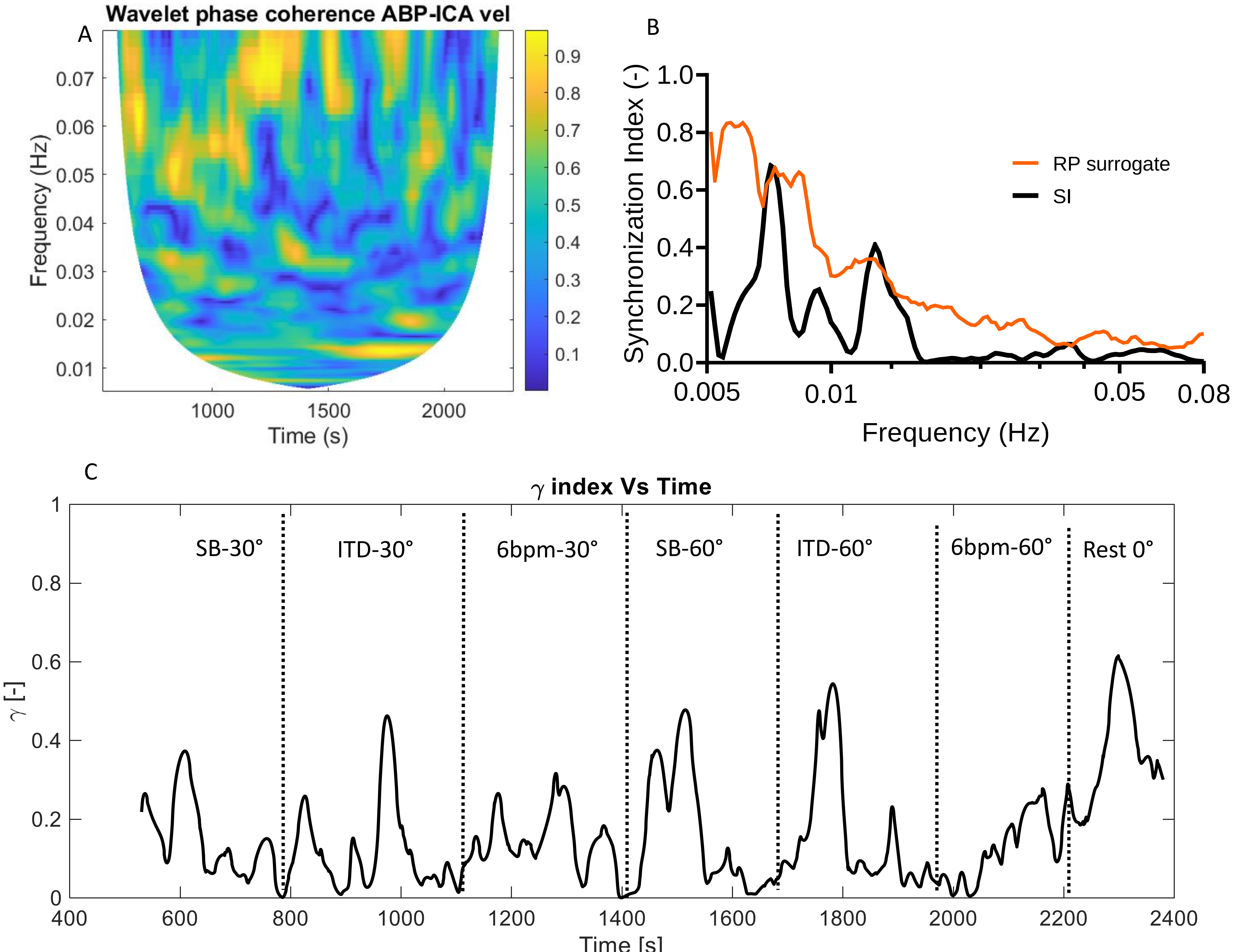

A
Wavelet phase coherence ABP-ICA vel
Frequency (Hz)
0.07
0.06
0.05
0.04
0.03
0.02
0.01
1000
1500
2000
Time (s)
0.9
0.8
0.7
0.6
0.5
0.4
0.3
0.2
0.1
B
Synchronization Index (-)
1.0
0.8
0.6
0.4
0.2
0.0
0.005
0.01
0.05
0.08
Frequency (Hz)
RP surrogate
SI
C
γ index Vs Time
γ [-]
1
0.8
0.6
0.4
0.2
0
400
600
800
1000
1200
1400
1600
1800
2000
2200
2400
Time [s]
SB-30°
ITD-30°
6bpm-30°
SB-60°
ITD-60°
6bpm-60°
Rest 0°

Subject 03

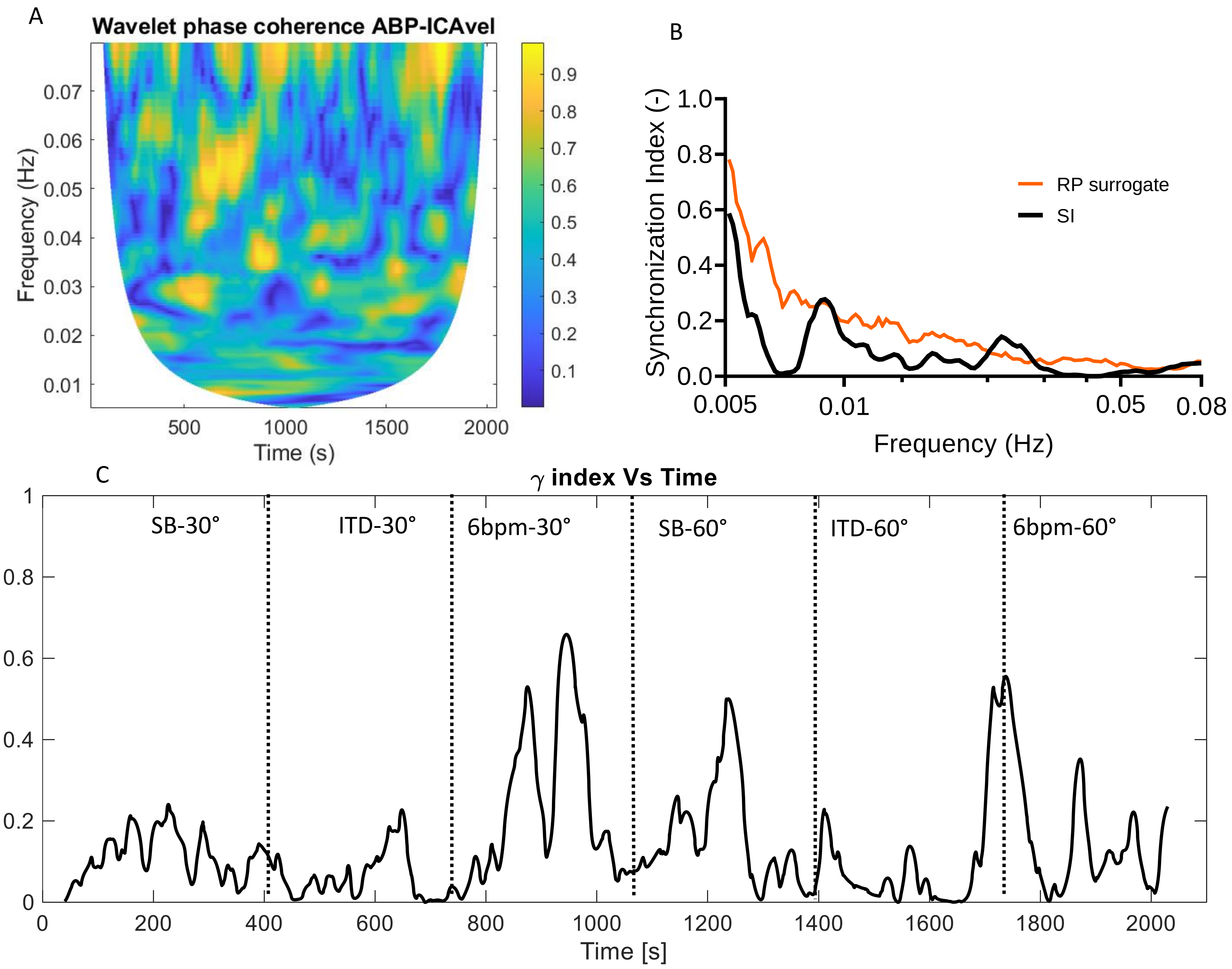
A
Wavelet phase coherence ABP-ICAvel
Frequency (Hz)
Time (s)
B
Synchronization Index (-)
RP surrogate
SI
Frequency (Hz)
C
γ index Vs Time
SB-30°
ITD-30°
6bpm-30°
SB-60°
ITD-60°
6bpm-60°
γ [-]
Time [s]

Subject 04

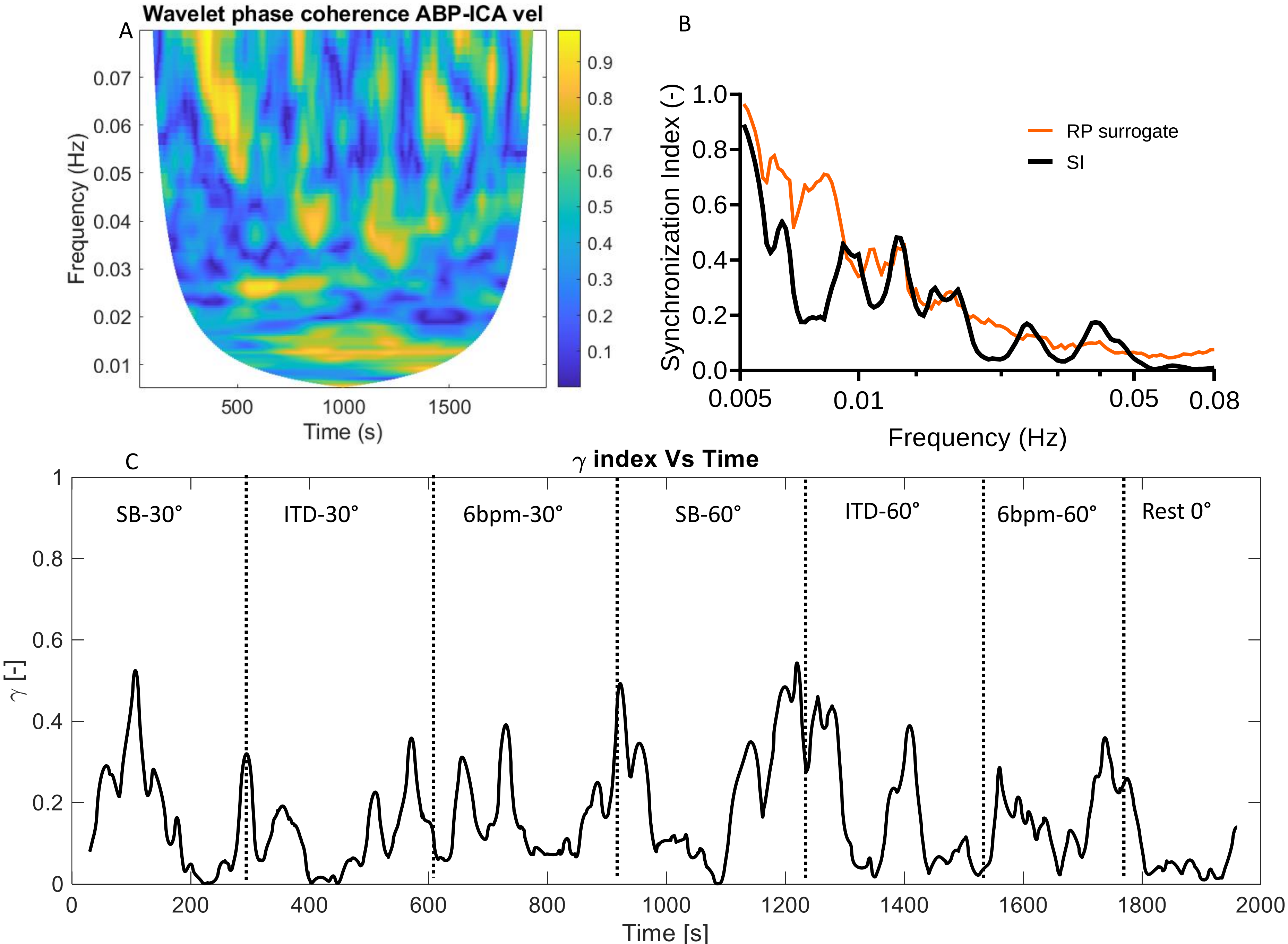

A
Wavelet phase coherence ABP-ICA vel
Frequency (Hz)
0.07
0.06
0.05
0.04
0.03
0.02
0.01
500
1000
1500
Time (s)
0.9
0.8
0.7
0.6
0.5
0.4
0.3
0.2
0.1
B
Synchronization Index (-)
1.0
0.8
0.6
0.4
0.2
0.0
0.005
0.01
0.05
0.08
Frequency (Hz)
RP surrogate
SI
C
γ index Vs Time
γ [-]
1
0.8
0.6
0.4
0.2
0
SB-30°
ITD-30°
6bpm-30°
SB-60°
ITD-60°
6bpm-60°
Rest 0°
0
200
400
600
800
1000
1200
1400
1600
1800
2000
Time [s]

Subject 05

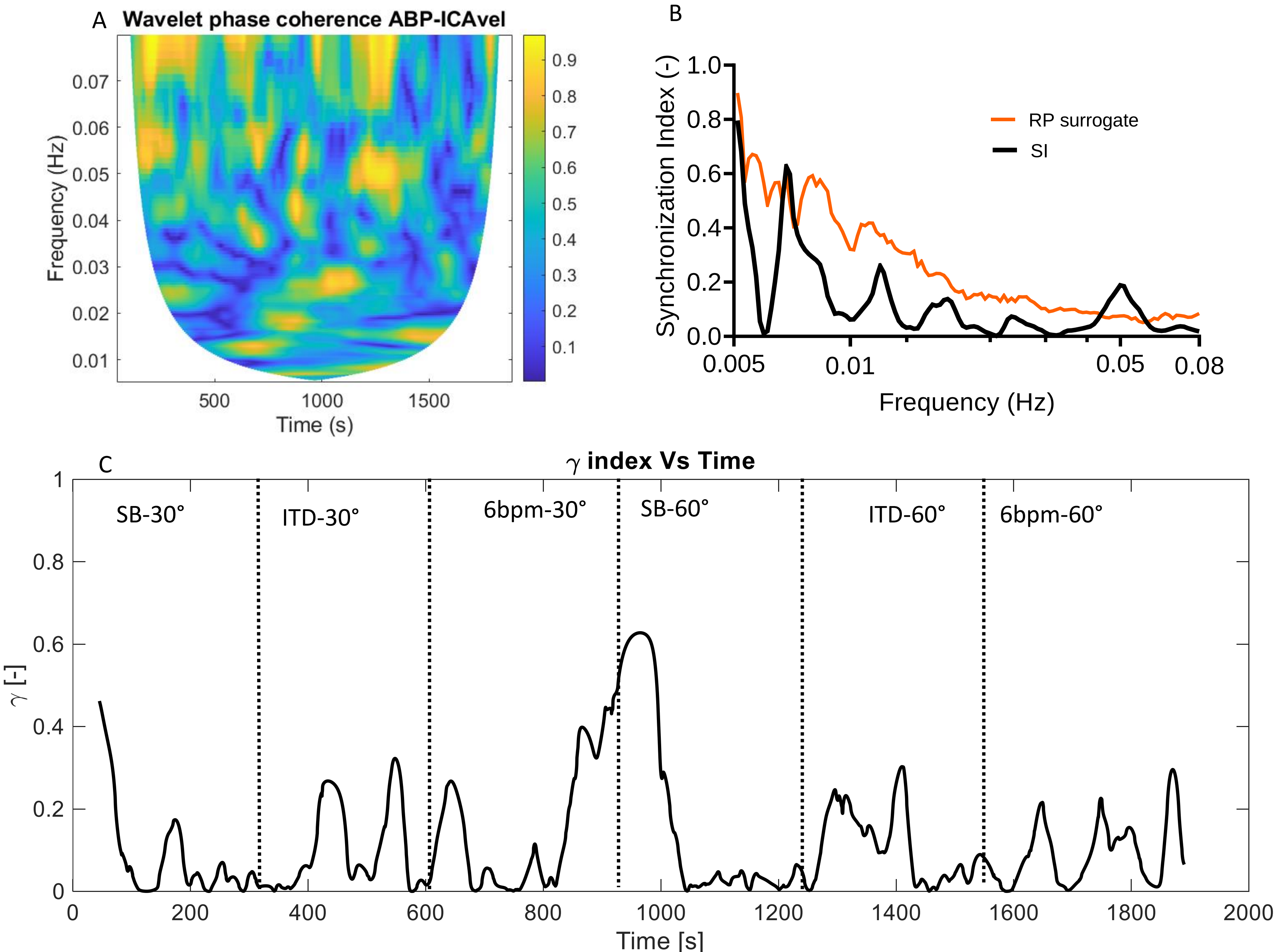
A
Wavelet phase coherence ABP-ICAvel
Frequency (Hz)
0.07
0.06
0.05
0.04
0.03
0.02
0.01
500
1000
1500
Time (s)
0.9
0.8
0.7
0.6
0.5
0.4
0.3
0.2
0.1
B
Synchronization Index (-)
1.0
0.8
0.6
0.4
0.2
0.0
RP surrogate
SI
0.005
0.01
0.05
0.08
Frequency (Hz)
C
γ index Vs Time
γ [-]
1
0.8
0.6
0.4
0.2
0
SB-30°
ITD-30°
6bpm-30°
SB-60°
ITD-60°
6bpm-60°
0
200
400
600
800
1000
1200
1400
1600
1800
2000
Time [s]

Subject 06

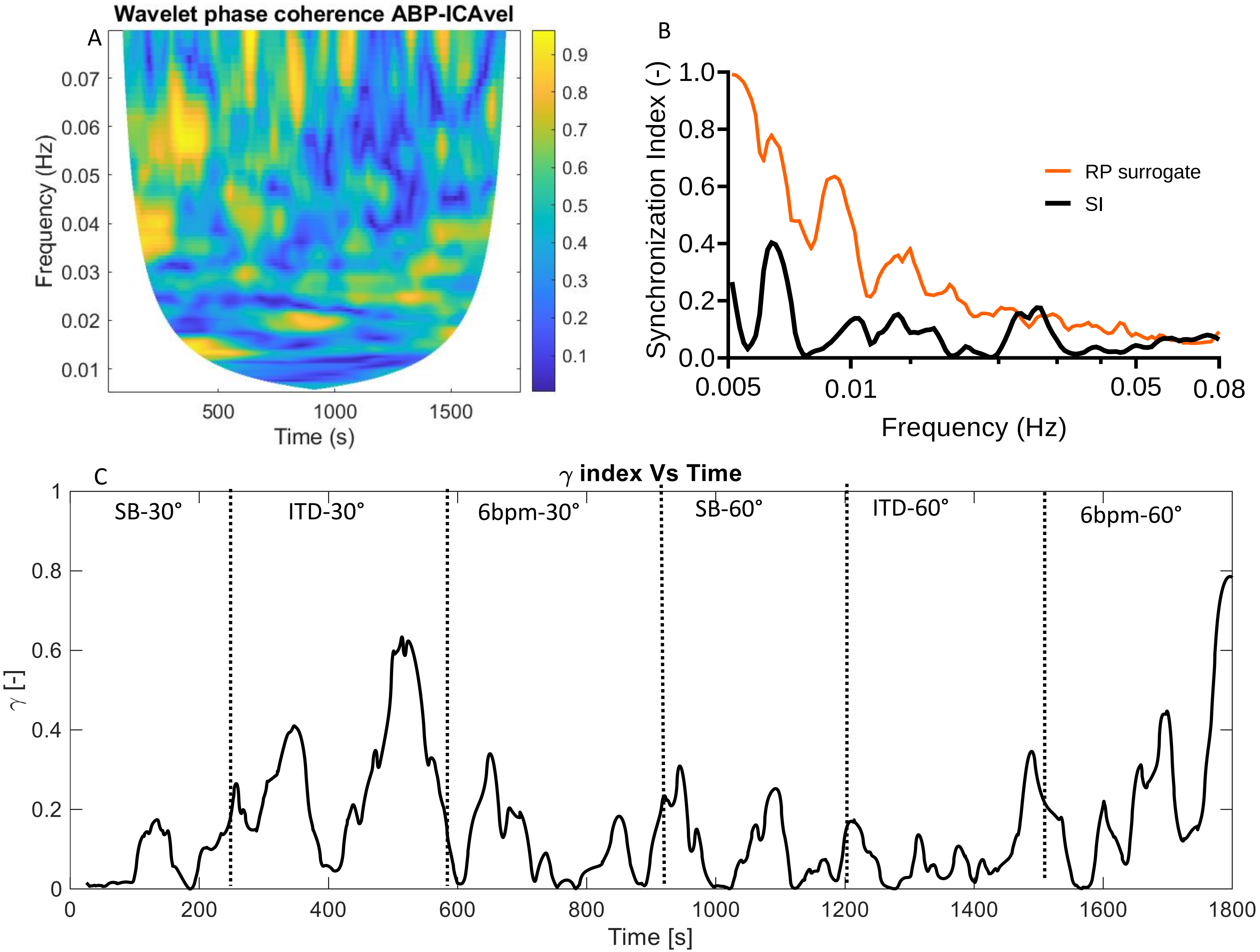

Wavelet phase coherence ABP-ICAvel
A
0.07
0.06
0.05
0.04
0.03
0.02
0.01
Frequency (Hz)
500
1000
1500
Time (s)
0.9
0.8
0.7
0.6
0.5
0.4
0.3
0.2
0.1
B
Synchronization Index (-)
1.0
0.8
0.6
0.4
0.2
0.0
0.005
0.01
0.05
0.08
Frequency (Hz)
RP surrogate
SI
C
γ index Vs Time
SB-30°
ITD-30°
6bpm-30°
SB-60°
ITD-60°
6bpm-60°
1
0.8
0.6
0.4
0.2
0
γ [-]
0
200
400
600
800
1000
1200
1400
1600
1800
Time [s]

Subject 07 (Figure 5)

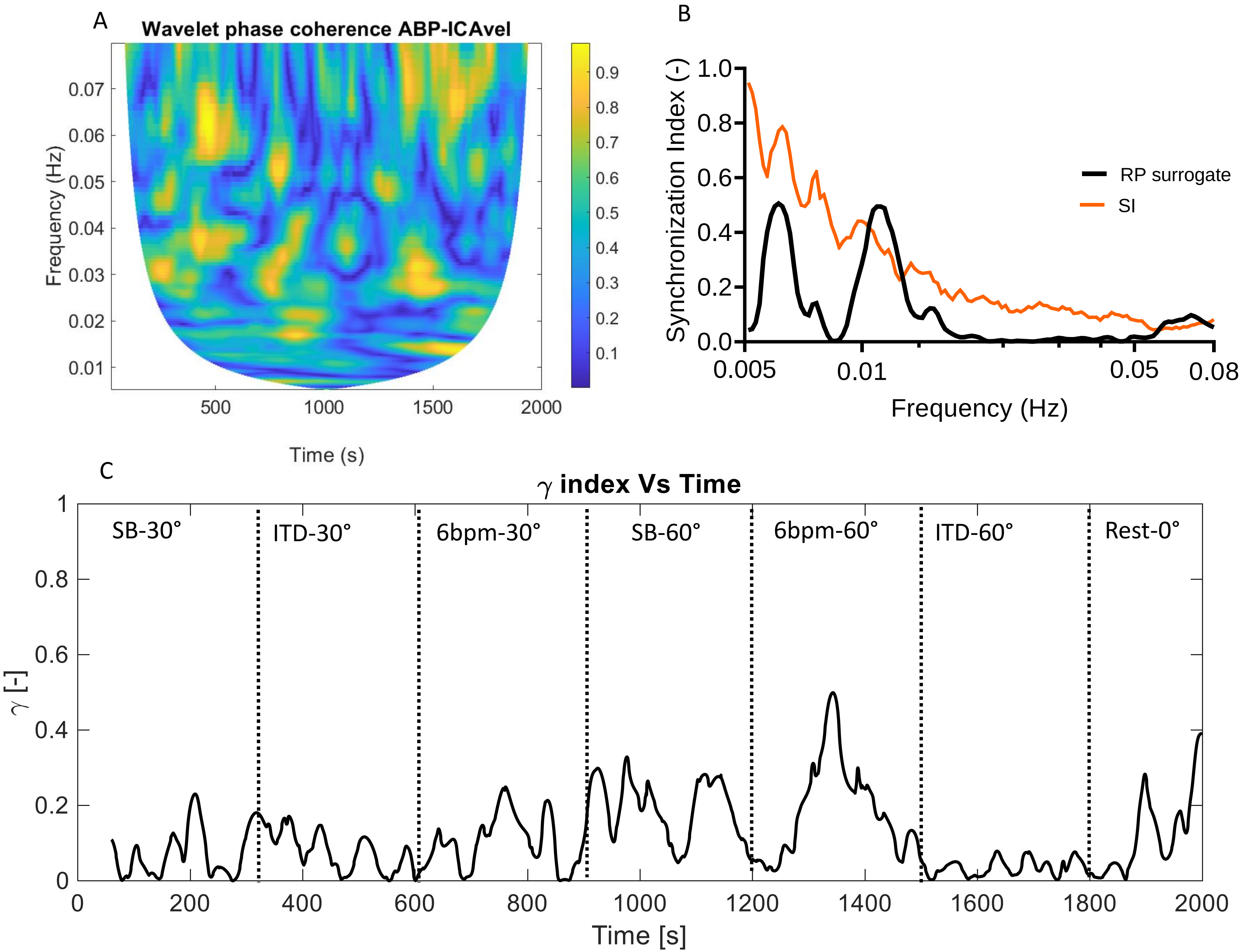

Subject 08

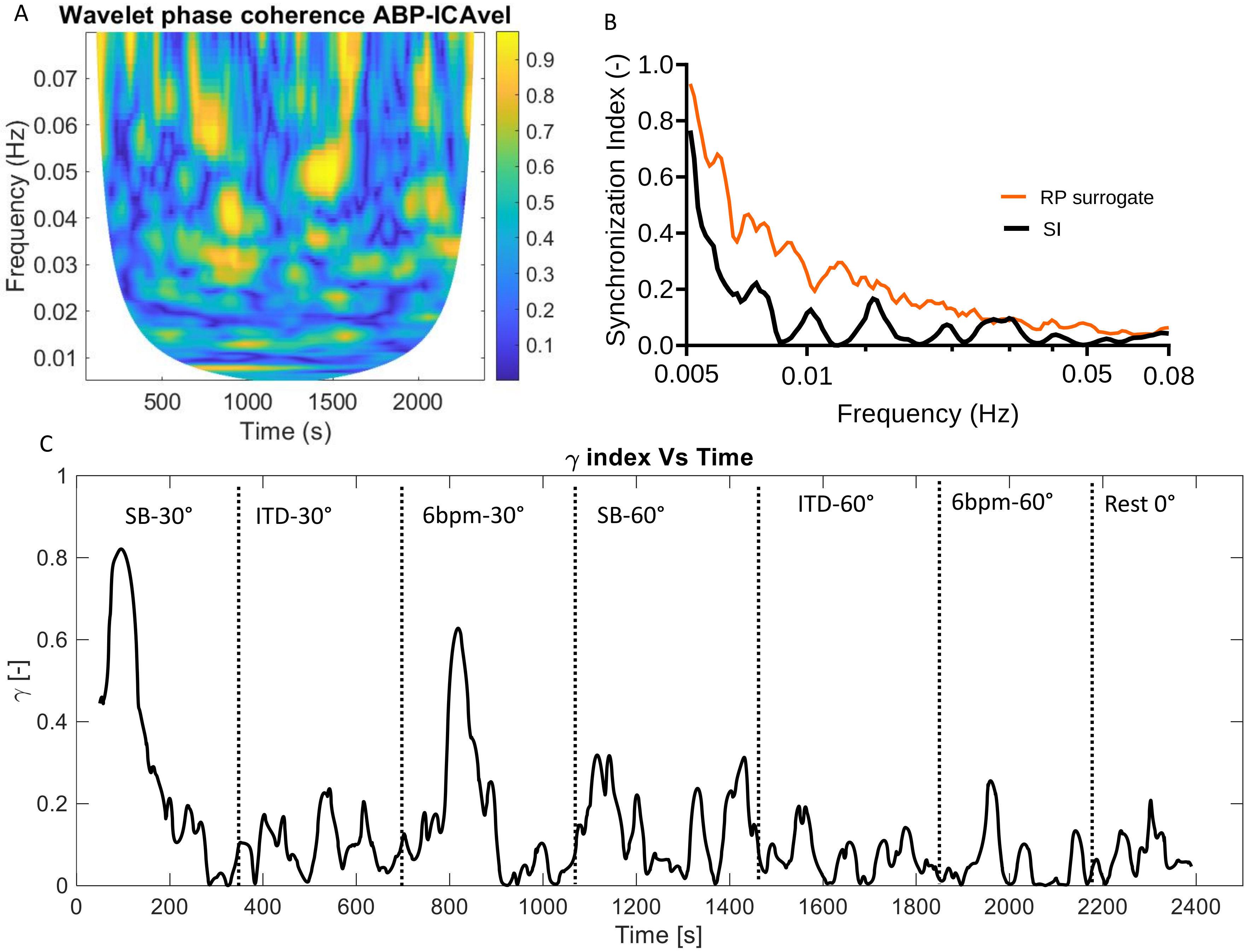

A
Wavelet phase coherence ABP-ICAvel
Frequency (Hz)
0.07
0.06
0.05
0.04
0.03
0.02
0.01
500
1000
1500
2000
Time (s)
0.9
0.8
0.7
0.6
0.5
0.4
0.3
0.2
0.1
B
Synchronization Index (-)
1.0
0.8
0.6
0.4
0.2
0.0
0.005
0.01
0.05
0.08
Frequency (Hz)
RP surrogate
SI
C
γ index Vs Time
γ [-]
1
0.8
0.6
0.4
0.2
0
SB-30°
ITD-30°
6bpm-30°
SB-60°
ITD-60°
6bpm-60°
Rest 0°
0
200
400
600
800
1000
1200
1400
1600
1800
2000
2200
2400
Time [s]

Subject 09

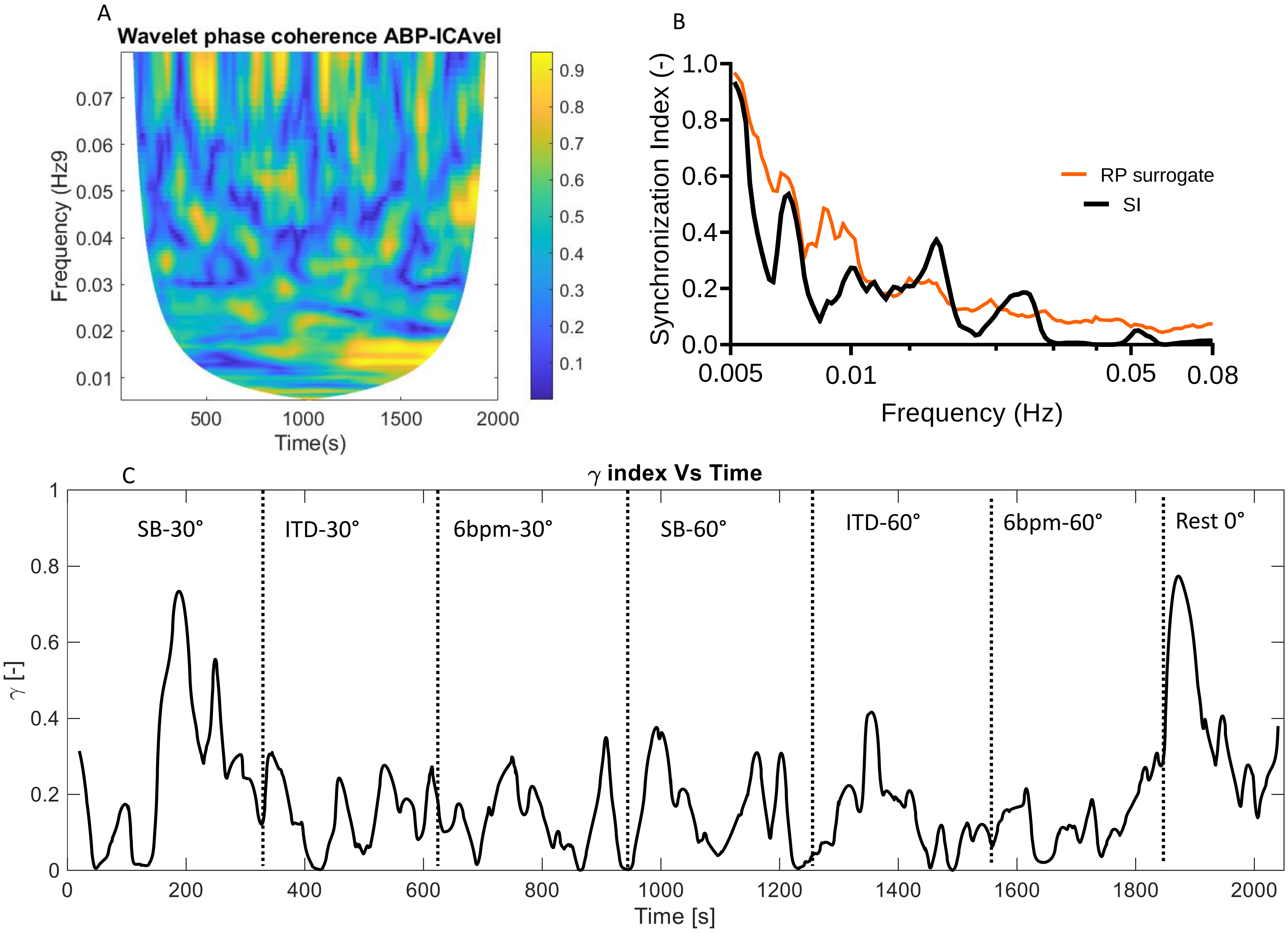
A
Wavelet phase coherence ABP-ICAvel
Frequency (Hz9
Time(s)
0.07
0.06
0.05
0.04
0.03
0.02
0.01
500
1000
1500
2000
0.9
0.8
0.7
0.6
0.5
0.4
0.3
0.2
0.1
B
Synchronization Index (-)
1.0
0.8
0.6
0.4
0.2
0.0
0.005
0.01
0.05
0.08
Frequency (Hz)
RP surrogate
SI
C
γ index Vs Time
γ [-]
1
0.8
0.6
0.4
0.2
0
SB-30°
ITD-30°
6bpm-30°
SB-60°
ITD-60°
6bpm-60°
Rest 0°
0
200
400
600
800
1000
1200
1400
1600
1800
2000
Time [s]

Subject 10

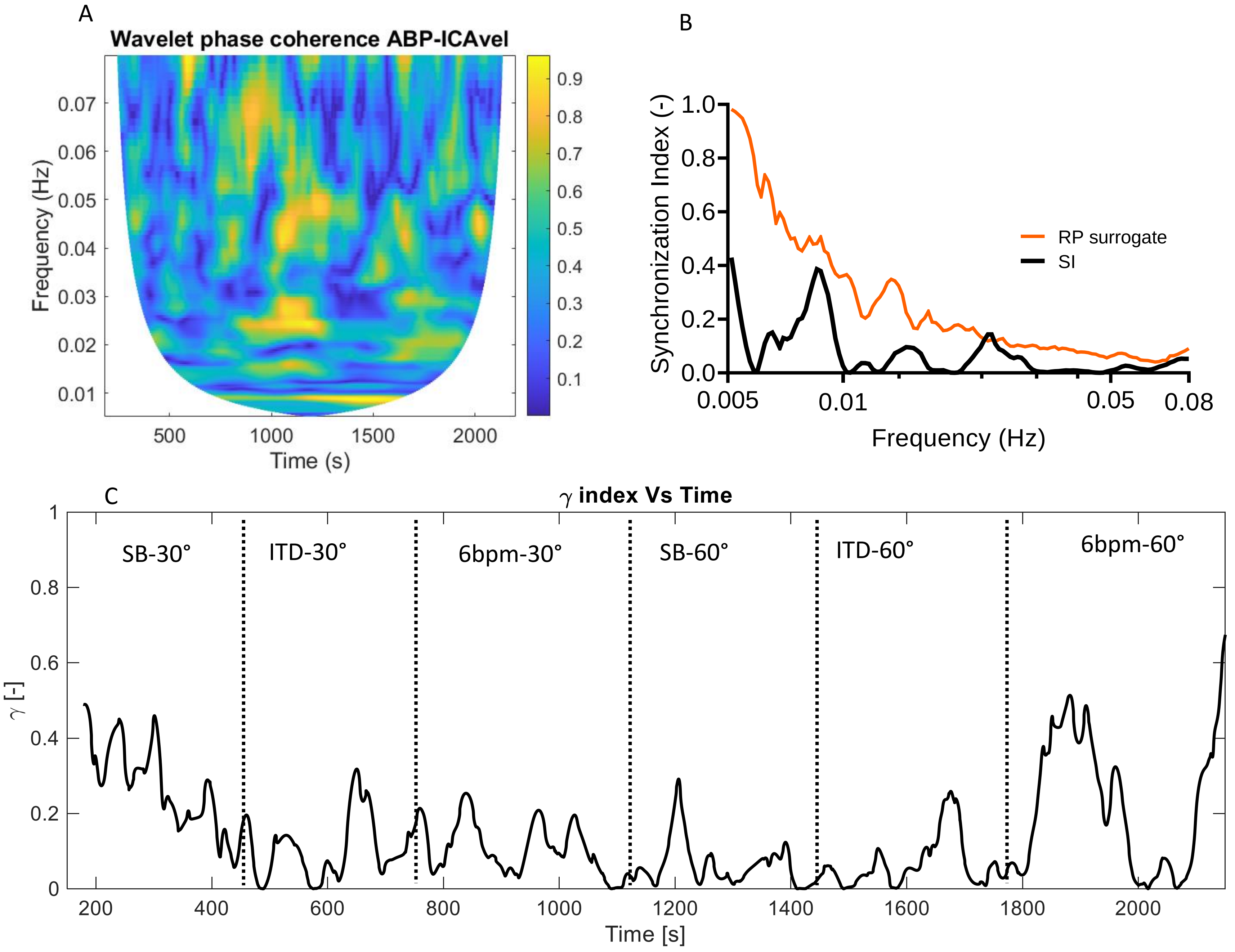

A
Wavelet phase coherence ABP-ICAvel
Frequency (Hz)
0.07
0.06
0.05
0.04
0.03
0.02
0.01
500
1000
1500
2000
Time (s)
0.9
0.8
0.7
0.6
0.5
0.4
0.3
0.2
0.1
B
Synchronization Index (-)
1.0
0.8
0.6
0.4
0.2
0.0
0.005
0.01
0.05
0.08
Frequency (Hz)
RP surrogate
SI
C
γ index Vs Time
γ [-]
1
0.8
0.6
0.4
0.2
0
SB-30°
ITD-30°
6bpm-30°
SB-60°
ITD-60°
6bpm-60°
200
400
600
800
1000
1200
1400
1600
1800
2000
Time [s]
