# Supplementary figures and images for: Dynamic cerebral autoregulation is preserved during orthostasis and intrathoracic pressure regulation in healthy subjects: A pilot study
Source: Physiol Rep. 2024 Apr 29;12(9):e16027. doi: 10.14814/phy2.16027 (PMC11058003; doi:10.14814/phy2.16027)

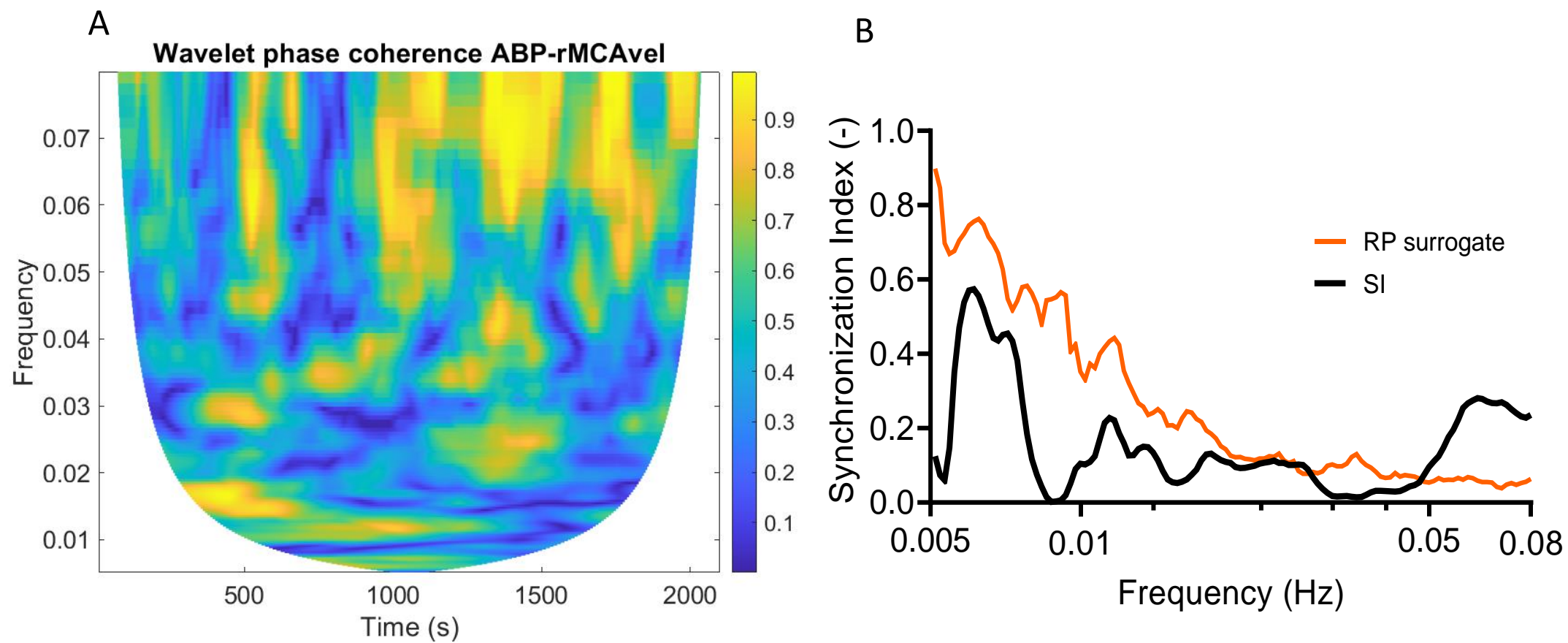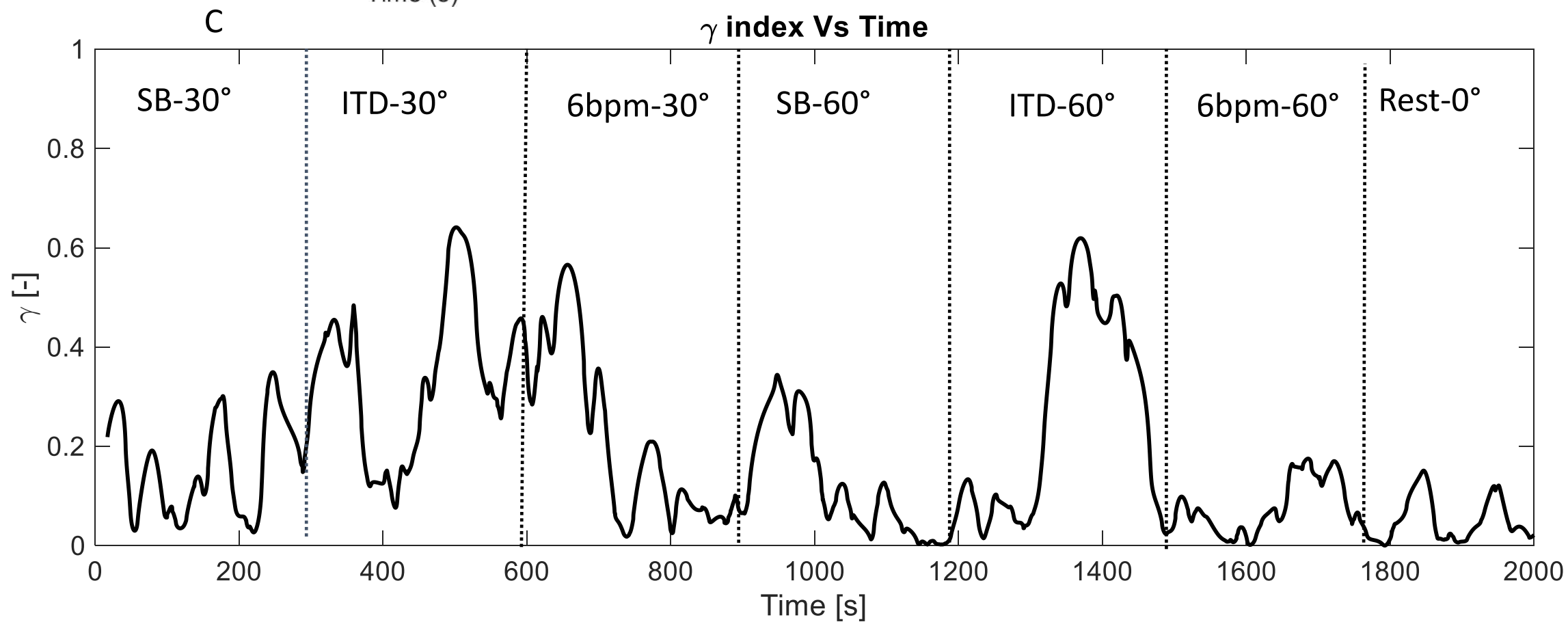

Supplement: Supplementary file 1 — Data S1: [file PHY2-12-e16027-s001.zip › PHYSREP-2024-01-021-T-s02.pdf]
